# Supplementary material for: Seladelpar improves liver stiffness in PBC: ASSURE interim analysis
Source: JHEP Rep. 2026 Oct 6;8(12):102057. doi: 10.1016/j.jhepr.2026.102057 (PMC13760463; doi:10.1016/j.jhepr.2026.102057)
Supplement: Supplementary file 1 [file mmc1.pdf]

Seladelpar improves liver stiffness in PBC: ASSURE interim analysis

Christopher L. Bowlus, Gideon M. Hirschfield, Alejandra M. Villamil, Kris V. Kowdley, Hany Elbeshbeshy, Christophe Corpechot, Andreas E. Kremer, Palak J. Trivedi, Yiannis Kallis, Sook-Hyang Jeong, Kyung min Kwon, Victor de Ledinghen, Xin Qi, Daria B. Crittenden, Cynthia Levy, on behalf of the ASSURE Investigators

Table of contents

Fig. S1 2

Fig. S2 4

Table S1 5

Table S2 6

Table S3 7

Table S4 8

Table S5 9

Table S6 10

Table S7 11

Table S8 12

Supplementary references 13

SUPPLEMENTARY FIGURES

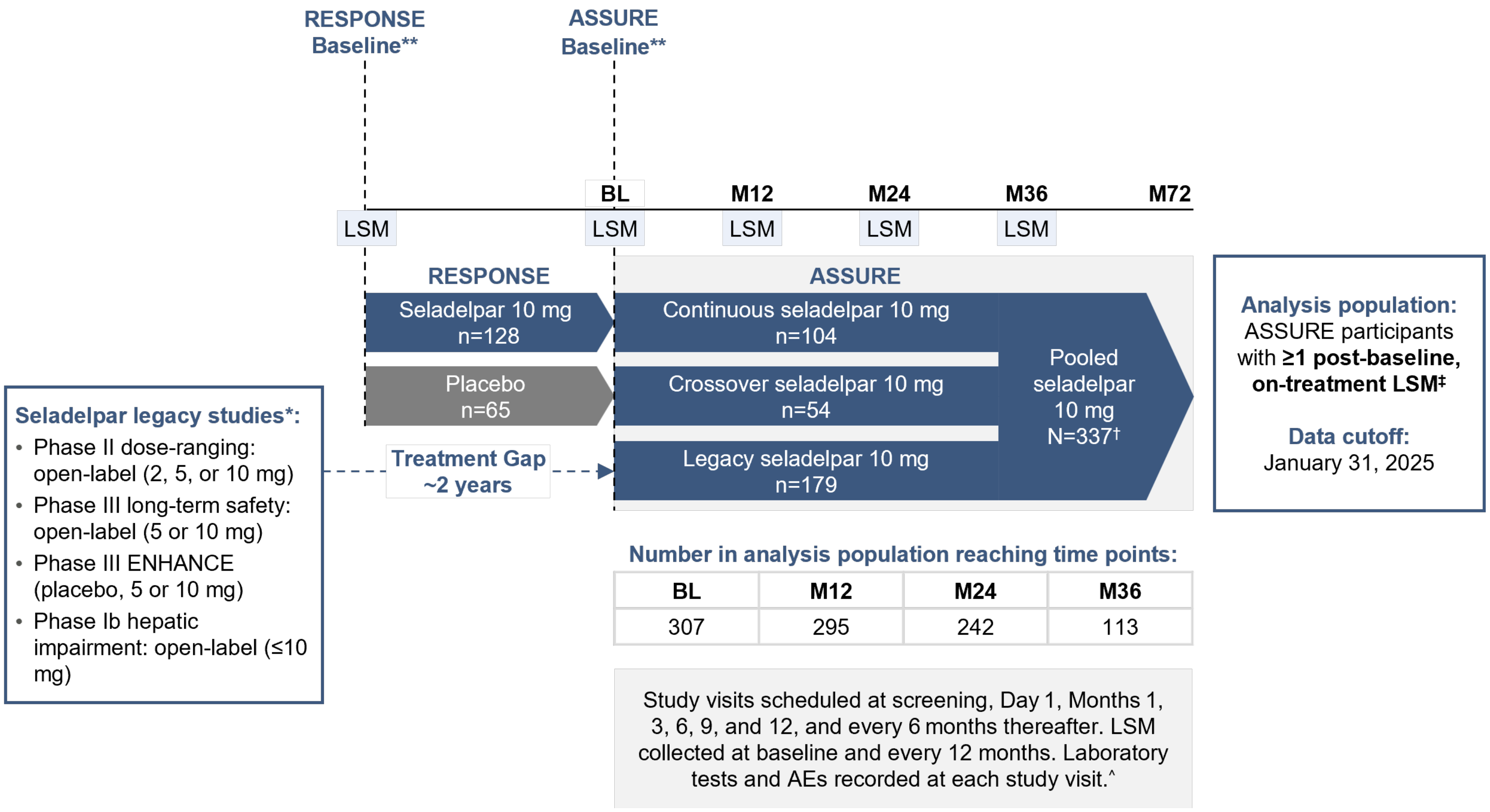

BL	M12	M24	M36
307	295	242	113

Fig. S1. ASSURE study schema.

*Legacy studies included the phase II dose-ranging study (NCT02955602),[1] phase III long-term safety study (NCT03301506), phase III ENHANCE study (NCT03602560),[2] and phase Ib hepatic impairment study (NCT04950764). The phase III placebo-controlled RESPONSE study (NCT04620733) and phase III, open-label ASSURE study (NCT03301506) are registered at clinicaltrials.gov. The phase II and III parent studies required an inadequate response or intolerance to first-line UDCA; **For patients who enrolled in ASSURE from the seladelpar arm in RESPONSE, BL was defined as seladelpar initiation in RESPONSE and RESPONSE data were included in the analysis. For patients who crossed over from the placebo arm of RESPONSE or had previously participated in seladelpar legacy studies, baseline was defined as seladelpar initiation in ASSURE; †Treatment gaps between legacy studies and ASSURE ranged from 27 days (phase Ib impairment study) to 212 weeks (phase III ENHANCE study); ‡In ASSURE, 2 patients initiated seladelpar at 5 mg and were excluded from the analysis; ^As part of data quality procedures, LSM values with ≥50% increase or ≥30% decrease from the prior measurement were queried for accuracy of LSM and IQR. A reliable LSM was defined by an IQR/median ratio ≤30%, and measurements that did not meet this threshold were excluded from the analysis; LSM was assessed in RESPONSE and ASSURE using VCTE and collected with local reads. Patients were instructed to fast for ≥4 hours prior to examination if possible. In RESPONSE, LSM was assessed at baseline and at Months 6 and 12; in ASSURE, LSM was assessed at screening and every 12 months thereafter. This analysis exclusively included LSMs collected using FibroScan® (Echosens, France). AE, adverse event; BL, baseline; IQR, interquartile range; LSM, liver stiffness measurement; M, month; UDCA, ursodeoxycholic acid; VCTE, vibration-controlled transient elastography.

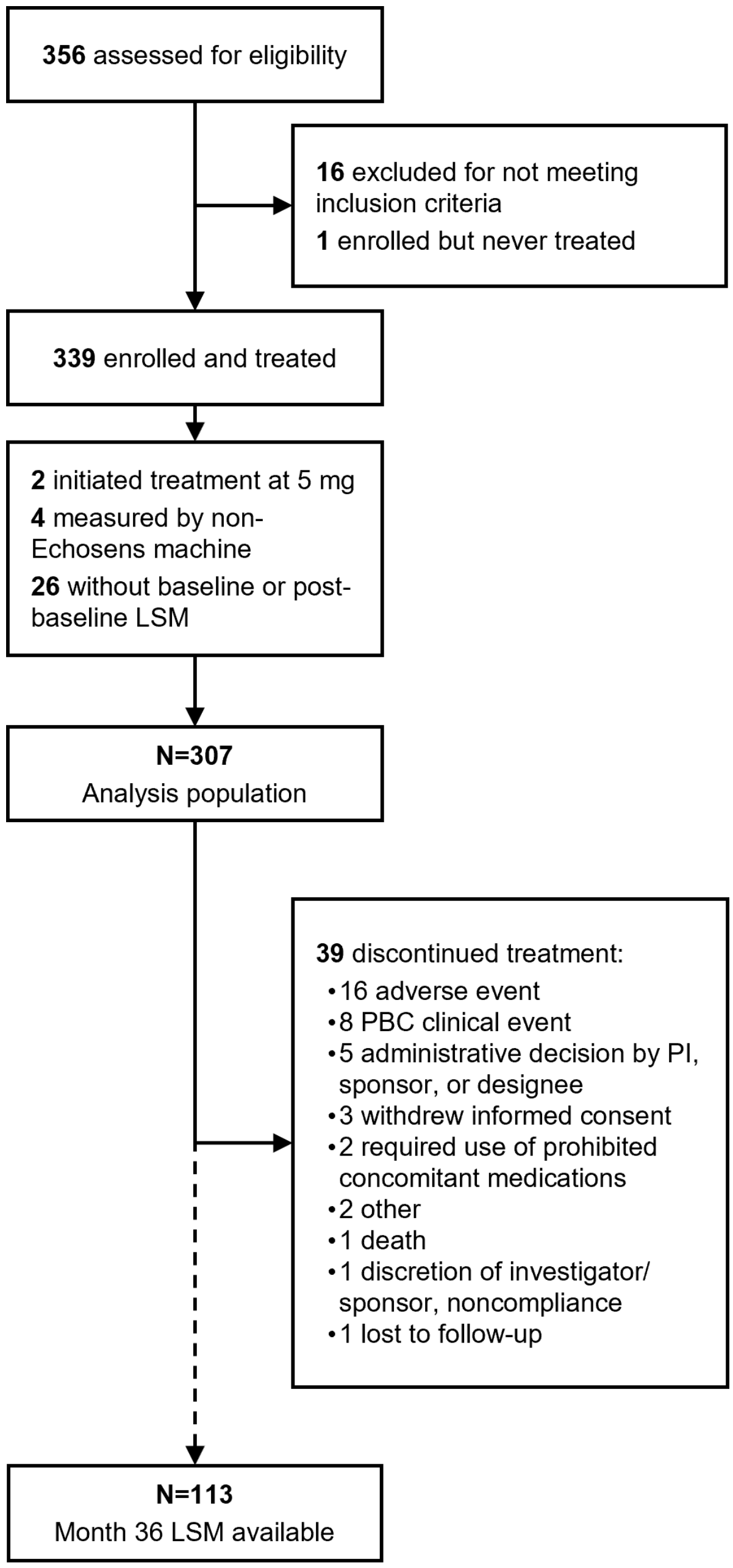

Fig. S2. ASSURE patient disposition for liver stiffness analysis population.

Interim analysis with data cutoff of January 31, 2025. In the analysis population, 145 patients rolled over to ASSURE from the phase III RESPONSE study and 162 enrolled from previous seladelpar legacy studies after meeting ASSURE eligibility criteria. ASSURE is an ongoing study and some patients had not yet reached Month 36 prior to the data cut-off. LSM, liver stiffness measurement; PBC, primary biliary cholangitis; PI, principal investigator.

SUPPLEMENTARY TABLES

Table S1. Baseline demographic and clinical characteristics in the overall population and by baseline Baveno VII category.

	Overall (N=307)	Baseline LSM <10 kPa* (N=209)	Baseline LSM ≥10 to <15 kPa* (N=57)	Baseline LSM ≥15 kPa* (N=41)
**Age (years), mean (SD)**	58.5 (9.6)	58.4 (9.3)	57.6 (11.0)	59.9 (9.5)
**Sex, n (%)**				
**Female**	290 (94.5)	200 (95.7)	55 (96.5)	35 (85.4)
**Male**	17 (5.5)	9 (4.3)	2 (3.5)	6 (14.6)
**BMI ≥30 (kg/m^2), n (%)**	85 (27.7)	54 (25.8)	19 (33.3)	12 (29.3)
**ALP (U/L), mean (SD)****	288.0 (128.5)	279.2 (116.9)	308.4 (153.8)	304.5 (144.4)
**Total bilirubin (mg/dL), mean (SD)**	0.7 (0.3)	0.7 (0.3)	0.8 (0.3)	1.0 (0.4)
**ALT (U/L), mean (SD)†**	42.8 (23.4)	40.8 (23.2)	48.0 (25.4)	46.0 (20.6)
**AST (U/L), mean (SD)‡**	37.9 (16.5)	35.0 (15.7)	43.6 (17.4)	44.5 (15.9)
**GGT (U/L), mean (SD)^**	227.9 (198.0)	209.7 (200.4)	258.1 (160.6)	278.3 (222.0)
**ALB (g/dL), mean (SD)**	4.1 (0.3)	4.2 (0.3)	4.1 (0.3)	4.0 (0.3)
**Platelet (10^3/uL), mean (SD)**	240.4 (79.0)	256.4 (75.0)	209.2 (80.0)	202.2 (73.3)
**INR, mean (SD)**	1.0 (0.1)	1.0 (0.1)	1.0 (0.1)	1.0 (0.1)
**LSM (kPa), mean (SD)**	9.8 (7.4)	6.5 (1.6)	11.9 (1.3)	23.9 (11.6)
**ELF score, mean (SD)**	10.0 (1.0)	9.7 (0.8)	10.6 (0.8)	11.0 (1.1)
**Cirrhosis: n (%)§**	49 (16.0)	8 (3.8)	10 (17.5)	31 (75.6)
**Child-Pugh-A, n (%)¶**	46 (93.9)	8 (100)	10 (100)	28 (90.3)
**Child-Pugh-B, n (%)¶**	3 (6.1)	0	0	3 (9.7)
**Child-Pugh score, mean (SD)¶**	5.2 (0.5)	5.1 (0.4)	5.0 (0.0)	5.3 (0.6)

*Baseline was defined as the time of seladelpar initiation (at entry to RESPONSE for patients randomized to seladelpar in RESPONSE, and at entry to ASSURE for all other patients); **The ULN for ALP is 116 U/L in men and women; †The ULN for ALT is 41 U/L in men and women; ‡The ULN for AST is 34 U/L in men and women; ^The ULN for GGT is 52 U/L in men and 38 U/L in women; §Cirrhosis at baseline was defined per-protocol and diagnostic criteria included liver biopsy, clinical history, laboratory findings, imaging findings, and/or clinical determination by the investigator. Full cirrhosis diagnosis criteria have been reported previously[3]; ¶Among patients with cirrhosis. ALB, albumin; ALP, alkaline phosphatase; ALT, alanine aminotransferase; AST, aspartate aminotransferase; BMI, body mass index; ELF, Enhanced Liver Fibrosis; GGT, gamma-glutamyl transferase; INR, international normalized ratio; LSM, liver stiffness measurement.

Table S2. Median LSM over time. (A) Median LSM in the overall population. (B) Median LSM by baseline LSM risk category.

A

	Overall Population			
	**Baseline (N=307)**	**Month 12 (N=295)**	**Month 24 (N=242)**	**Month 36 (N=113)**
**LSM (kPa), median (Q1, Q3)**	7.5 (5.9, 11.2)	7.7 (5.6, 11.4)	7.6 (5.9, 11.6)	7.8 (5.6, 10.7)
**LSM (kPa), absolute change from BL, median (Q1, Q3)**	—	0 (−1.3, 1.4)	0.1 (−1.4, 2.1)	−0.3 (−1.7, 1.8)
**% LSM change from BL, median (Q1, Q3)**	—	0 (−16.9, 19.8)	1.7 (−17.0, 30.0)	−3.4 (−22.8, 24.5)

B

	Low Risk Baseline LSM <10.7 kPa				Intermediate Risk Baseline LSM ≥10.7 to <16.9 kPa				High Risk Baseline LSM ≥16.9 kPa			
	**Baseline (N=223)**	**Month 12 (N=216)**	**Month 24 (N=184)**	**Month 36 (N=88)**	**Baseline (N=51)**	**Month 12 (N=47)**	**Month 24 (N=34)**	**Month 36 (N=14)**	**Baseline (N=33)**	**Month 12 (N=32)**	**Month 24 (N=24)**	**Month 36 (N=11)**
**LSM (kPa) median (Q1, Q3)**	6.6 (5.5, 8.0)	6.4 (5.2, 8.3)	6.7 (5.4, 8.7)	6.6 (5.0, 9.0)	12.6 (11.6, 13.6)	13.6 (11.1, 15.7)	13.6 (10.1, 17.3)	11.5 (10.3, 17.2)	21.1 (18.9, 28.4)	20.6 (13.4, 36.2)	15.8 (13.7, 33.8)	14.3 (10.0, 21.3)
**LSM (kPa), absolute change from BL, median (Q1, Q3)**	—	0 (−1.1, 1.2)	0.2 (−0.9, 1.5)	0.1 (−1.4, 1.8)	—	0 (−1.6, 2.7)	0.7 (−2.9, 4.7)	−0.9 (−3.6, 4.8)	—	−2.8 (−7.2, 4.5)	−3.2 (−6.6, 5.9)	−5.2 (−10.6, 2.4)
**% LSM change from BL, median (Q1, Q3)**	—	0 (−15.1, 21.8)	2.7 (−11.9, 27.0)	1.8 (−18.6, 26.0)	—	0 (−12.5, 16.7)	4.8 (−21.3, 37.9)	−7.4 (−29.5, 38.7)	—	−13.1 (−27.1, 17.5)	−17.8 (−29.0, 30.4)	−29.7 (−51.5, 12.7)

An increase in liver stiffness from baseline ≥30% at any timepoint was considered clinically meaningful worsening, while a decrease in liver stiffness from baseline ≥30% was considered clinically meaningful improvement. BL, baseline; LSM, liver stiffness measurement; Q1, first quartile; Q3, third quartile.

Table S3. Median LSM over time by Baveno VII category.

	Baseline LSM <10 kPa				Baseline LSM ≥10 to <15 kPa				Baseline LSM ≥15 kPa			
	**Baseline (N=209)**	**Month 12 (N=202)**	**Month 24 (N=173)**	**Month 36 (N=82)**	**Baseline (N=57)**	**Month 12 (N=53)**	**Month 24 (N=39)**	**Month 36 (N=15)**	**Baseline (N=41)**	**Month 12 (N=40)**	**Month 24 (N=30)**	**Month 36 (N=16)**
**LSM (kPa) median (Q1, Q3)**	6.5 (5.4, 7.6)	6.2 (5.1, 8.0)	6.6 (5.4, 8.1)	6.4 (4.9, 8.3)	11.9 (10.8, 12.8)	12.0 (9.9, 14.4)	12.0 (9.6, 16.9)	11.2 (9.0, 16.3)	19.8 (17.5, 26.8)	16.4 (13.4, 27.2)	15.8 (13.7, 30.3)	14.5 (10.2, 21.2)
**LSM (kPa), absolute change from BL, median (Q1, Q3)**	—	0.0 (−1.0, 1.1)	0.1 (−0.9, 1.4)	0.1 (−1.4, 1.7)	—	0.2 (−1.5, 2.8)	0.9 (−2.6, 4.8)	−0.5 (−1.5, 4.8)	—	−1.9 (−7.1, 2.8)	−3.0 (−5.2, 5.5)	−3.9 (−6.2, 1.5)
**% LSM change from BL, median (Q1, Q3)**	—	0.0 (−15.9, 21.7)	2.0 (−11.8, 26.4)	1.6 (−19.2, 25.9)	—	1.5 (−12.5, 24.4)	8.7 (−20.5, 43.4)	−4.1 (−11.8, 38.7)	—	−9.7 (−27.1, 16.0)	−16.6 (−28.2, 24.8)	−22.0 (−37.3, 7.9)

BL, baseline; LSM, liver stiffness measurement; Q1, first quartile; Q3, third quartile.

Table S4. Characteristics of patients with ≥30% worsening and high-risk category at Month 36.

	Patient 1	Patient 2	Patient 3	Patient 4	Patient 5	Patient 6
**Baseline characteristics**						
**Age (years)**	46	55	49	63	66	55
**Age at PBC diagnosis (years)**	39	34	43	53	56	51
**Sex**	Female	Female	Female	Female	Female	Female
**ALP (U/L)***	223.0	349.5	230.0	183.0	324.3	647.0
**Total bilirubin (mg/dL)**	0.4	1.4	0.5	0.7	1.2	0.9
**LSM (kPa)**	8.7	15.4	12.6	12.4	29.8	11.1
**Cirrhosis****	No	No	Yes	No	Yes	No
**Child-Pugh, class (score)**	—	—	Child-Pugh-A (5)	—	Child-Pugh-A (5)	—
**On-treatment characteristics**						
**M12 CBR**	No	No	No	Yes	No	No
**M12 ALP ≤ULN**	No	No	No	Yes	No	No
**M36 ALP % change from baseline**	−11.7	−22.2	−37.0	−62.8	−40.8	−53.2
**M36 LSM (kPa)**	25.7	33.6	25.3	17.2	40.2	50.1

*The ULN for ALP is 116 U/L in men and women; **Cirrhosis at baseline was defined per-protocol and diagnostic criteria included liver biopsy, clinical history, laboratory findings, imaging findings, and/or clinical determination by the investigator. Full cirrhosis diagnosis criteria have been reported previously.[3] ALP, alkaline phosphatase; CBR, composite biochemical response; LSM, liver stiffness measurement; M, month; PBC, primary biliary cholangitis; ULN, upper limit of normal.

Table S5. Univariate analysis for factors associated with LSM stability or improvement versus ≥20% worsening from baseline at Month 36.

	<20% worsening or any improvement (N=78)	≥20% Worsening (N=35)	p value
**Baseline characteristics**			
**Age (years), mean (SD)**	60.0 (9.5)	57.4 (8.8)	0.1876
**Age at PBC diagnosis (years), mean (SD)**	49 (8.3)	48 (7.8)	0.8133
**Female, n (%)**	73 (93.6)	33 (94.3)	0.8876
**BMI ≥30 (kg/m^2), n (%)**	30 (38.5)	8 (22.9)	0.1060
**LSM (kPa), mean (SD)****	9.3 (5.2)	8.3 (4.9)	0.4177
**≥10 kPa, n (%)**	23 (29.5)	8 (22.9)	0.4671
**ELF, mean (SD)**	9.7 (0.8)	9.7 (0.9)	0.7562
**AST (U/L), mean (SD)**	33.1 (11.4)	39.4 (14.9)	0.0225*
**ALT (U/L), mean (SD)**	38.7 (18.0)	47.5 (24.5)	0.0832
**ALP (U/L), mean (SD)**	250.5 (81.5)	304.6 (116.8)	0.0353*
**<1.5× ULN, n (%)**	16 (20.5)	1 (2.9)	0.0157*
**Total bilirubin (mg/dL), mean (SD)**	0.7 (0.2)	0.8 (0.3)	0.0172*
**≤ULN, n (%)**	74 (94.9)	29 (82.9)	0.0384*
**<0.6× ULN, n (%)**	47 (60.3)	12 (34.3)	0.0110*
**GGT (U/L), mean (SD)**	204.4 (215.6)	232.3 (153.7)	0.0883
**ALB (g/dL), mean (SD)**	4.2 (0.3)	4.2 (0.3)	0.7325
**Platelet (10^3/uL), mean (SD)**	242.8 (80.9)†	238.6 (53.6)	0.8689
**Cirrhosis, n (%)**	13 (16.7)	3 (8.6)	0.2559
**On-treatment biochemical response at Month 12**			
**CBR, n (%)**	70 (89.7)	21 (60.0)	0.0002*
**ALP <1.67× ULN, n (%)**	72 (92.3)	24 (68.6)	0.0012*
**ALP ≥15% reduction, n (%)**	77 (98.7)	31 (88.6)	0.0158*
**Total bilirubin ≤ULN, n (%)**	76 (97.4)	32 (91.4)	0.1529
**ALP <1.5× ULN, n (%)**	68 (87.2)	22 (62.9)	0.0031*
**ALP ≤ULN, n (%)**	39 (50.0)	11 (31.4)	0.0673

Statistical differences were assessed using a two-sided Wilcoxon rank sum test for continuous variables and a Cochran-Mantel-Haenszel test for general association for categorical variables, performed for each factor in each comparison. *p<0.05; **Baseline LSM (kPa) thresholds evaluated for statistical significance in the univariate analysis included: <10.7, ≥16.9, and an among-groups comparison of <10.7 vs. ≥10.7 to <16.9 vs. ≥16.9; none reached significance at a level of p<0.05; †Assessed in N=76 patients. ALB, albumin; ALP, alkaline phosphatase; ALT, alanine aminotransferase; AST, aspartate aminotransferase; BMI, body mass index; CBR, composite biochemical response; ELF, Enhanced Liver Fibrosis; GGT, gamma-glutamyl transferase; LSM, liver stiffness measurement; PBC, primary biliary cholangitis; ULN, upper limit of normal.

Table S6. Univariate analysis for factors associated with no LSM increase versus any increase from baseline at Month 36.

	No increase (≤0%) (N=59)	Any increase (>0%) (N=54)	p value
**Baseline characteristics**			
Age (years), mean (SD)	59.0 (9.6)	59.4 (9.1)	0.9381
Age at PBC diagnosis (years), mean (SD)	48 (8.4)	50 (7.7)	0.2171
Female, n (%)	55 (93.2)	51 (94.4)	0.7884
BMI ≥30 (kg/m^2), n (%)	24 (40.7)	14 (25.9)	0.0988
LSM (kPa), mean (SD)**	9.8 (5.5)	8.2 (4.5)	0.0771
≥10 kPa, n (%)	19 (32.2)	12 (22.2)	0.2370
ELF, mean (SD)	9.8 (0.9)	9.5 (0.8)	0.1002
AST (U/L), mean (SD)	33.8 (11.6)	36.3 (14.1)	0.3838
ALT (U/L), mean (SD)	39.8 (18.7)	43.2 (22.4)	0.5215
ALP (U/L), mean (SD)	244.8 (83.8)	291.8 (104.3)	0.0161*
<1.5× ULN, n (%)	13 (22.0)	4 (7.4)	0.0306*
Total bilirubin (mg/dL), mean (SD)[†]	0.6 (0.2)	0.8 (0.3)	0.0477*
GGT (U/L), mean (SD)	193.4 (195.4)	234.5 (200.9)	0.0556
ALB (g/dL), mean (SD)	4.2 (0.3)	4.2 (0.2)	0.1170
Platelet (10^3/uL), mean (SD)	237.6 (82.1)[‡]	245.8 (62.4)[^]	0.5931
Cirrhosis, n (%)	9 (15.3)	7 (13.0)	0.7283
**On-treatment biochemical response at Month 12**			
CBR, n (%)	55 (93.2)	36 (66.7)	0.0004*
ALP <1.67× ULN, n (%)	55 (93.2)	41 (75.9)	0.0105*
ALP ≥15% reduction, n (%)	58 (98.3)	50 (92.6)	0.1420
Total bilirubin ≤ULN, n (%)	59 (100)	49 (90.7)	0.0173*
ALP <1.5× ULN, n (%)	51 (86.4)	39 (72.2)	0.0619
ALP ≤ULN, n (%)	30 (50.8)	20 (37.0)	0.1416

Statistical differences were assessed using a two-sided Wilcoxon rank sum test for continuous variables and a Cochran-Mantel-Haenszel test for general association for categorical variables, performed for each factor in each comparison. *p<0.05; **Baseline LSM (kPa) thresholds evaluated for statistical significance in the univariate analysis included: <10.7, ≥16.9, and an among-groups comparison of <10.7 vs. ≥10.7 to <16.9 vs. ≥16.9; none reached significance at a level of p<0.05; [†]Total bilirubin ≤ULN and <0.6× ULN were additionally analyzed and did not reach significance; [‡]Assessed in N=58 patients; [^]Assessed in N=53 patients. ALB, albumin; ALP, alkaline phosphatase; ALT, alanine aminotransferase; AST, aspartate aminotransferase; BMI, body mass index; CBR, composite biochemical response; ELF, Enhanced Liver Fibrosis; GGT, gamma-glutamyl transferase; LSM, liver stiffness measurement; PBC, primary biliary cholangitis; ULN, upper limit of normal.

Table S7. Odds of LSM stability or improvement versus ≥20% worsening at Month 36 by biochemical response at Month 12.

	N	<20% worsening or any improvement (N=78)	≥20% Worsening (N=35)
**Biochemical response at Month 12, n (%)**			
**ALP normalization**	50	39 (78.0)	11 (22.0)
**CBR with ALP >ULN**	43	32 (74.4)	11 (25.6)
**CBR non-response (reference)***	20	7 (35.0)	13 (65.0)
**Odds ratio for LSM stability/improvement** at Month 36 (95% CI)**			
**ALP normalization[†]**	—	6.58 (2.11, 20.52)	
**CBR with ALP >ULN[†]**	—	5.40 (1.72, 17.00)	

A logistic regression model with achievement of biochemical response at Month 12 as the fixed effect was used for comparison. *This group included patients who did not achieve CBR or ALP normalization; **Stability/improvement defined as <20% worsening or any improvement in LSM; [†]Compared with CBR non-response reference group; ALP, alkaline phosphatase; CBR, composite biochemical response; LSM, liver stiffness measurement; ULN, upper limit of normal.

Table S8. Odds of no LSM increase versus any increase from baseline at Month 36.

	N	No increase (≤0%) (N=59)	Any increase (>0%) (N=54)
**Biochemical response at Month 12, n (%)**			
**ALP normalization**	50	30 (60.0)	20 (40.0)
**CBR with ALP >ULN**	43	25 (58.1)	18 (41.9)
**CBR non-response (reference)***	20	4 (20.0)	16 (80.0)
**Odds ratio for LSM stability/improvement** at Month 36 (95% CI)**			
**ALP normalization[†]**	—	6.00 (1.75, 20.59)	
**CBR with ALP >ULN[†]**	—	5.56 (1.59, 19.43)	

A logistic regression model with achievement of biochemical response at Month 12 as the fixed effect was used for comparison. *This group included patients who did not achieve CBR or ALP normalization; **Stability/improvement defined as no increase in LSM; [†]Compared with CBR non-response reference group. ALP, alkaline phosphatase; CBR, composite biochemical response; LSM, liver stiffness measurement; ULN, upper limit of normal.

SUPPLEMENTARY REFERENCES

1. Bowlus CL, Galambos MR, Aspinall RJ, et al. A phase II, randomized, open-label, 52-week study of seladelpar in patients with primary biliary cholangitis. J Hepatol 2022;77(2):353–364.
2. Hirschfield GM, Shiffman ML, Gulamhusein A, et al. Seladelpar efficacy and safety at 3 months in patients with primary biliary cholangitis: ENHANCE, a phase 3, randomized, placebo-controlled study. Hepatology 2023;78(2):397–415.
3. Levy C, Trivedi PJ, Kowdley KV, et al. Long-term efficacy and safety of selective PPARδ agonist seladelpar in primary biliary cholangitis: ASSURE interim study results. Am J Gastroenterol 2025;121(5):1140–1153 [Suppl. Epub ahead of print].
