# Supplementary material for: Seladelpar improves liver stiffness in PBC: ASSURE interim analysis
Source: JHEP Rep. 2026 Oct 6;8(12):102057. doi: 10.1016/j.jhepr.2026.102057 (PMC13760463; doi:10.1016/j.jhepr.2026.102057)
Supplement: Supplementary file 3 [file mmc4.pdf]

Research article

JHEP Reports

Seladelpar improves liver stiffness in PBC: ASSURE interim analysis

Authors

Christopher L. Bowlus, Gideon M. Hirschfield, Alejandra M. Villamil, …, Xin Qi, Daria B. Crittenden, Cynthia Levy

Correspondence

clevy@med.miami.edu (C. Levy).

Graphical abstract

Objectives

- To explore LSM changes over time and the associations between biochemical response and LSM stability or improvement over 3 years of seladelpar treatment

Study population

- Patients in ASSURE with ≥1 post-baseline, on-treatment LSM (VCTE) as of January 31, 2025
- The analysis included 307 patients; 113 patients had LSM data available through month 36

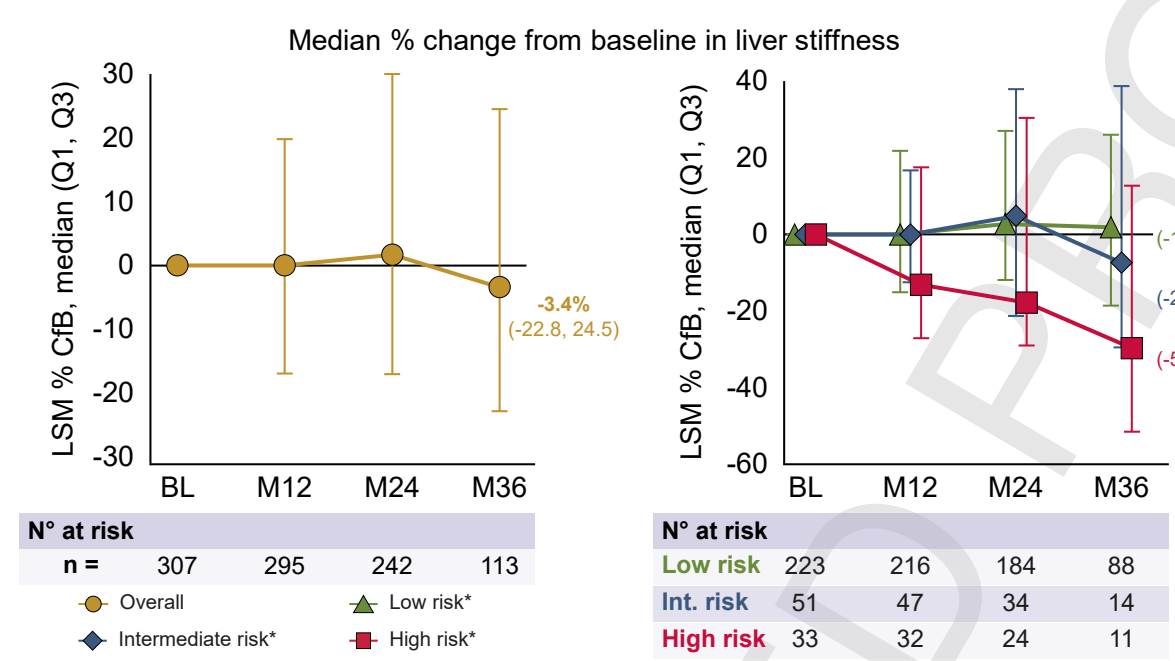

- Most patients (89/113; 78.8%) on seladelpar who were evaluable at month 36 had stable/improved LSM

Conclusions

- Most patients with 3-year LSMs available demonstrated stable or improved liver stiffness with seladelpar treatment
- Patients with high baseline LSM showed LSM reductions at 3 years
- Maintenance or improvement of liver stiffness was associated with biochemical response

Criteria for worsening	Biochemical response at M12	Responder n/N (%)	OR for LSM stability improvement *vs.* worsening at M36 (95% CI)**
≥30%	CBR† with ALP >UL	36/43 (83.7)	4.21 (1.27, 13.92)
	ALP normalization	42/50 (84.0)	4.30 (1.35, 13.71)
≥20%	CBR† with ALP >ULN	32/43 (74.4)	5.40 (1.72, 17.00)
	ALP normalization	39/50 (78.0)	6.58 (2.11, 20.52)
>0%	CBR† with ALP >ULN	25/43 (58.1)	5.56 (1.59, 19.43)
	ALP normalization	30/50 (60.0)	6.00 (1.75, 20.59)

*Patients were grouped by baseline LSM (kPa) into low (<10.7), intermediate (≥10.7 to <16.9), and high (≥16.9) risk; **Compared with patients who did not achieve CBR; †CBR defined as ALP <1.67× ULN, total bilirubin ≤ULN, and an ALP decrease ≥15% from BL.
ALP, alkaline phosphatase; BL, baseline; CBR, composite biochemical response; CfB, change from baseline; CI, confidence interval; int., intermediate; LSM, liver stiffness measurement; M, month; OR, odds ratio; Q1, first quartile; Q3, third quartile; ULN, upper limit of normal; VCTE, vibration-controlled transient elastography.

Q1 ## Highlights:

- Liver stiffness was stable or improved with seladelpar in the majority of patients with PBC who had 3-year data available.
- With up to 3 years of seladelpar treatment, liver stiffness decreased in patients at high risk of disease progression.
- Biochemical response was associated with stable or improved liver stiffness.

Impact and implications:

Liver stiffness measurement (LSM) by vibration-controlled transient elastography is a prognostic tool reflecting risk of disease progression in patients with primary biliary cholangitis. Among patients with baseline liver stiffness (FibroScan) ≥10 kPa, those with cirrhosis, and those who do not receive adequate treatment, LSM has been observed to increase over time. This interim analysis suggests that long-term treatment with seladelpar, an approved second-line therapy, may lead to stable or improved LSM, with biochemical response associated with LSM stability and improvement. These results build on existing evidence of the efficacy of seladelpar, suggesting its potential to favorably impact disease progression. The results also highlight the importance of alkaline phosphatase reduction to reduce risk of disease progression in patients with primary biliary cholangitis.

https://doi.org/10.1016/j.jhepr.2026.102057

© 2026 Gilead Sciences, Inc. Published by Elsevier B.V. on behalf of European Association for the Study of the Liver (EASL). This is an open access article under the CC BY license (http://creativecommons.org/licenses/by/4.0/).

UNCORRECTED PROOF

ARTICLE IN PRESS

Research article

JHEP|Reports

Q11 Seladelpar improves liver stiffness in PBC: ASSURE interim analysis

Q10 Christopher L. Bowlus[1], Gideon M. Hirschfield[2], Alejandra M. Villamil[3], Kris V. Kowdley[4], Hany Elbeshbeshy[5], Christophe Corpechot[6], Andreas E. Kremer[7], Palak J. Trivedi[8,9], Yiannis Kallis[10], Sook-Hyang Jeong[11], Kyung min Kwon[12,†], Victor de Ledinghen[13], Xin Qi[12], Daria B. Crittenden[12], Cynthia Levy[14,*], on behalf of the ASSURE Investigators

JHEP Reports **2026**. vol. ■ | 1–10

Background & Aims: Liver stiffness measurement (LSM) by vibration-controlled transient elastography can predict clinical outcomes in patients with primary biliary cholangitis (PBC), with ongoing disease activity associated with progressive LSM increases. Seladelpar is a first-in-class delpar approved for the treatment of PBC that improves biochemical markers and symptoms. We assessed changes in LSM with up to 3 years of seladelpar treatment in an interim analysis of the ongoing, open-label ASSURE study.

Methods: Patients with PBC rolled over into ASSURE from the pivotal, placebo-controlled RESPONSE study or enrolled after participation in earlier legacy studies. Patients who received seladelpar 10 mg with ≥1 on-treatment LSM were evaluated (cut-off: January 31, 2025). Patients were grouped by baseline LSM (kPa) into low-risk (<10.7), intermediate-risk (≥10.7 to <16.9), and high-risk (≥16.9) categories. LSM change from baseline was evaluated overall and by baseline risk category, with category shifts assessed at Month (M)36. Multivariate analysis identified factors associated with stable/improved *vs.* worsening LSM at M36.

Results: A total of 307 patients were included, with baseline mean (SD) LSM (kPa) of 9.8 (7.4); 72.6% were in the low-risk, 16.6% in the intermediate-risk, and 10.7% in the high-risk category. Among 113 patients with M36 LSMs, median change from baseline was –3.4% overall and –29.7% among high-risk patients. Most patients (89/113; 78.8%) had stable/improved LSM at M36. Biochemical response at M12 was significantly associated with LSM stability/improvement at M36 (odds ratio [95% CI] 4.30 [1.35, 13.71] for alkaline phosphatase [ALP] normalization and 4.21 [1.27, 13.92] for ALP >ULN but <1.67× ULN *vs.* ALP ≥1.67 × ULN).

Conclusions: Most patients with M36 LSMs available showed stable/improved liver stiffness with seladelpar treatment, with reductions observed among high-risk patients. Maintenance or improvement of liver stiffness was associated with biochemical response.

Clinical trial number: NCT03301506.

© 2026 Gilead Sciences, Inc. Published by Elsevier B.V. on behalf of European Association for the Study of the Liver (EASL). This is an open access article under the CC BY license (http://creativecommons.org/licenses/by/4.0/).

Introduction

Primary biliary cholangitis (PBC) is a chronic autoimmune cholestatic liver disease that can progress to cirrhosis and liver failure, with ursodeoxycholic acid (UDCA) as the first-line standard of care.[1,2] Alkaline phosphatase (ALP) and total bilirubin have been established as key predictors of clinical outcomes in PBC, including liver transplantation and death.[2,3] Using treatment response criteria of ALP <1.67× or <1.5× the upper limit of normal (ULN), up to 40% of patients have an inadequate biochemical response to UDCA, and about 70% have elevated ALP on UDCA monotherapy.[2,4,5] More recently, ALP normalization (ALP ≤ULN) has been suggested as a treatment goal for PBC, with any elevation of ALP above normal shown to increase the risk of liver transplantation or death.[6,7]

In addition to biochemical markers, liver stiffness measurement (LSM) assessed by vibration-controlled transient elastography (VCTE) has been shown to predict clinical outcomes in patients with PBC.[8,9] Increased liver stiffness has been consistently associated with a higher risk of liver decompensation, liver transplantation, and death.[6,10] Recent data suggest that a value >10 kPa on a patient's most recent LSM is associated with a significantly higher risk of hepatic decompensation. In that study, patients with PBC and advanced chronic liver disease (LSM ≥10 kPa) treated with UDCA, obeticholic acid, or fibrates also experienced a median increase in LSM of 3.5 kPa over a median of 47 months.[11] In patients with cirrhosis treated with UDCA monotherapy, annual LSM increases of 4.1 kPa have been observed over a 5-year period.[9]

* Corresponding author. Address: Division of Digestive Health and Liver Diseases, University of Miami School of Medicine, 1120 NW 14th Street, Suite 1284, Miami, Florida 33136, USA.
E-mail address: clevy@med.miami.edu (C. Levy).
† Affiliation at the time research was conducted.
https://doi.org/10.1016/j.jhepr.2026.102057

UNCORRECTED PROOF

Seladelpar is a first-in-class delpar (selective peroxisome proliferator-activated receptor delta [PPAR-δ] agonist) indicated for the treatment of PBC in combination with UDCA in adults who have an inadequate response to UDCA, or as monotherapy in patients who are unable to tolerate UDCA.[12–14] Seladelpar reduces bile acid synthesis and has demonstrated anti-cholestatic, anti-inflammatory, anti-fibrotic, and anti-pruritic effects in human and animal studies.[15–17] In the pivotal phase III RESPONSE trial, a significantly greater proportion of patients treated with seladelpar 10 mg achieved the composite biochemical response (CBR; defined as ALP <1.67× ULN, total bilirubin ≤ULN, and an ALP reduction of ≥15% from baseline) and ALP normalization compared with placebo after 1 year of treatment. Seladelpar also significantly improved pruritus in patients who had moderate-to-severe pruritus at baseline. There were no meaningful changes in LSM in the seladelpar or placebo groups over the 1-year treatment period of RESPONSE, and long-term LSM trends with seladelpar treatment have not yet been established.[17] To address this, we evaluated LSM over time in patients treated with seladelpar for up to 3 years in an interim analysis of the ongoing, long-term, open-label ASSURE study, including an exploration of the association of biochemical response with stability or improvement in LSM.

Patients and methods

Study design and population

ASSURE (NCT03301506) is an ongoing, 5-year, open-label, uncontrolled phase III study of seladelpar in patients with PBC, conducted at 88 international sites. Patients who completed the pivotal 12-month, placebo-controlled phase III RESPONSE trial (NCT04620733) could roll over directly into ASSURE. Patients who participated in earlier legacy seladelpar studies could also enroll in ASSURE if they met the ASSURE eligibility criteria. Detailed study design and participation criteria have been previously described.[15,18] This analysis included pooled ASSURE patients receiving seladelpar 10 mg who had ≥1 post-baseline, on-treatment LSM at scheduled visits (Months 12, 24, and 36) with a data cut-off of January 31, 2025.

Baseline was defined at seladelpar initiation in ASSURE or, if relevant, RESPONSE. For patients who enrolled in ASSURE from the seladelpar arm of RESPONSE, baseline was defined as seladelpar initiation in RESPONSE, and RESPONSE data were included in this analysis. For patients who crossed over from the placebo arm of RESPONSE or had previously participated in seladelpar legacy studies, baseline was defined as seladelpar initiation in ASSURE. LSM was assessed as an exploratory measure in RESPONSE and ASSURE using VCTE and collected with local reads. Patients were instructed to fast for ≥4 hours prior to examination if possible. In RESPONSE, LSM was assessed at baseline and at Months 6 and 12; in ASSURE, LSM was assessed at screening and every 12 months thereafter. This analysis exclusively included LSM collected using FibroScan® (Echosens, France). As part of data quality procedures, LSM values with ≥50% increase or ≥30% decrease from the prior measurement were queried for accuracy of LSM and IQR. A reliable LSM was defined by an IQR/median ratio ≤30%, and measurements that did not meet this threshold were excluded from the analysis. Cirrhosis at baseline was defined per-protocol and diagnostic criteria included liver biopsy, clinical history, laboratory findings, imaging findings, and/or clinical determination by the investigator. Full cirrhosis diagnostic criteria have been reported previously.[18,19] Laboratory tests and adverse events were recorded at each study visit. See study schema in Fig. S1 for additional details.

This study was conducted in accordance with ethical principles originating in the Declaration of Helsinki, Good Clinical Practice guidelines, and all applicable national and local laws and regulations.[20] The protocol was approved by each site's Institutional Review Board or Independent Ethics Committee, and all patients provided written informed consent.

Analyses of liver stiffness

In this analysis, changes in LSM (kPa) from baseline in the overall population were assessed at Months 12, 24, and 36. LSM thresholds were used to group patients by baseline LSM into the following three risk categories: low (LSM <10.7 kPa), intermediate (LSM ≥10.7 to <16.9 kPa), and high (LSM ≥16.9 kPa). These categories represent LSM-based risk stratification using thresholds derived from previously reported PBC-specific cut-offs shown to correlate with histologic fibrosis stage.[9] Additional exploratory analyses were conducted grouping patients by baseline LSM into the following three risk categories, based on thresholds identified by the Baveno VII workshop: LSM <10 kPa, LSM ≥10 to <15 kPa, and LSM ≥15 kPa.[21] Among patients who had reached Month 36 as of the data cut-off date, shifts in risk category from baseline were assessed at Month 36. A threshold of ≥30% change from baseline (either worsening or improvement) was used to define clinically meaningful change based on literature in chronic liver disease and initial real-world data from PBC.[10,21,22] Additional threshold cut-offs of ≥20% and >0% change were also explored, given the uncertainty around clinically relevant thresholds for LSM changes in PBC.

Patients who had LSM stability/improvement were compared with those who experienced ≥30% worsening to identify differences in their baseline characteristics and achievement of on-treatment biochemical response at Month 12 using univariate and multivariate analyses. We explored the association between LSM stability/improvement and achievement of ALP normalization *vs.* CBR with ALP >ULN at Month 12, compared with those who did not achieve CBR at Month 12. Additional analyses were conducted using worsening thresholds of ≥20% and >0% change from baseline. Percent change from baseline in ALP at Month 12 was also assessed as a continuous predictor of percent change from baseline in LSM at Month 36. Safety data from ASSURE, with a data cut-off of January 31, 2024, have been previously reported.[18]

All analyses here are considered exploratory and were conducted *post hoc*.

Statistical analysis

For patients who initiated seladelpar in RESPONSE, baseline was determined as the average of screening and Day 1 values in RESPONSE. For patients in the RESPONSE placebo arm who initiated seladelpar in ASSURE, baseline value was defined as the RESPONSE Month 12 value, unless additional screening and/or Day 1 laboratory assessments were collected

UNCORRECTED PROOF

in ASSURE. In those cases, baseline was defined as the average of values collected after the Month 12 assessment and before the first dose in ASSURE. For legacy patients who enrolled in ASSURE, baseline laboratory values were determined by the average of all measurements recorded during screening and prior to seladelpar initiation. Categorical variables were reported as number and percentage. Continuous variables were reported using descriptive statistics including mean, SD, SE, median, first and third quartiles (Q1, Q3), minimum, and maximum. Missing data were not imputed for summary statistics, and group denominators reflected available data at the specified timepoint. For biochemical response analyses, a patient was considered evaluable at a given timepoint if the analysis timepoint was scheduled to occur prior to the data cut-off data, regardless of the availability of biochemical assessment results. Evaluable patients with missing data were considered non-responders at the specified timepoint if they discontinued treatment due to adverse events, occurrence of a PBC clinical outcome, or use of prohibited concomitant medications.

Characteristics associated with LSM stability/improvement *vs.* ≥30%, ≥20%, and >0% worsening at Month 36 were evaluated using a two-sided Wilcoxon rank-sum test for continuous variables and, for categorical variables, a Cochran-Mantel-Haenszel test for general association, applied separately to each factor. Variables with p <0.05 were entered into multivariate logistic regression models using stepwise variable selection to identify the most informative predictors. For each variable retained, the final models reported odds ratios (ORs) with 95% CIs and p values. Separate logistic regressions compared patients who achieved biochemical response (CBR with ALP >ULN and ALP normalization) with patients who did not achieve CBR. Pearson's correlation analysis was used to evaluate the relationship between continuous changes from baseline in ALP and in LSM. Analyses were not adjusted for multiple comparisons. All analyses were performed using SAS software version 9.4 (SAS Institute, Cary, NC).

Results

Patient population and baseline characteristics

The overall evaluable population included 307 patients receiving seladelpar 10 mg with baseline and ≥1 post-baseline LSM value. Patient attrition for the liver stiffness analysis population as of January 31, 2025, is provided in Fig. S2. Overall, the mean (SD) age was 58.5 (9.6) years, and 290 patients (94.5%) were female. At baseline, mean (SD) liver stiffness was 9.8 (7.4) kPa, and 49 patients (16.0%) had cirrhosis, of whom 46 (93.9%) had Child-Pugh class A disease.

Among patients grouped by baseline LSM risk category, 223 (72.6%) patients were at low risk (LSM <10.7 kPa), 51 (16.6%) were at intermediate risk (LSM ≥10.7 to <16.9 kPa), and 33 (10.7%) were at high risk (LSM ≥16.9 kPa). In the high-risk group, a higher proportion of patients were male, and most patients (78.8%) had cirrhosis. Baseline ALP was generally similar across risk categories. Total bilirubin and aspartate aminotransferase were moderately higher, and albumin moderately lower, in the higher-risk categories. ELF (enhanced liver fibrosis) score increased with risk category (Table 1). Generally similar trends were observed when grouping patients by baseline LSM according to Baveno VII categories (Table S1).

Impact of seladelpar on liver stiffness through Month 36

Median values, absolute changes from baseline, and percentage changes from baseline in LSM over time for all evaluable ASSURE patients, both overall and by baseline LSM risk category, are shown in Fig. 1. Among the 113 patients Q3 treated with seladelpar who had available LSM values through Month 36, median (Q1, Q3) LSM at Month 36 was 7.8 (5.6, 10.7) kPa, representing a median (Q1, Q3) absolute change from baseline of –0.3 (–1.7, 1.8) kPa and median (Q1, Q3) percentage change from baseline of –3.4% (–22.8, 24.5; Fig. 1A, Table S2A). When evaluated by baseline LSM risk category, high-risk patients had a median (Q1, Q3) absolute change from baseline of –5.2 (–10.6, 2.4) kPa and median (Q1, Q3) percent change from baseline of –29.7% (–51.5, 12.7). Patients in the intermediate and low-risk groups showed stable LSM, with median (Q1, Q3) absolute and percentage changes from baseline of –0.9 (–3.6, 4.8) kPa and –7.4% (–29.5, 38.7) for the intermediate-risk group and 0.1 (–1.4, 1.8) kPa and 1.8% (–18.6, 26.0) for the low-risk group, respectively (Fig. 1B, Table S2B). Similar trends were also observed when patients were grouped by baseline Baveno VII category (Table S3). When exploring shifts based on LSM-based risk categories among the 113 patients with Month 36 LSM values, 97 patients (85.8%) remained in the same risk category or moved to a lower-risk category. This included 85 patients (75.2%) with no change in risk category and 12 patients (10.6%) who shifted to a lower-risk category. In contrast, 16/113 patients (14.2%) moved to a higher-risk category (Fig. 2).

Patients with clinically meaningful (≥30%) change in liver stiffness

At Month 36, 70/113 patients (61.9%) had stable LSM (within ±30% of baseline). An additional 19/113 patients (16.8%) had clinically meaningful improvements in LSM (≥30% decrease), and 24/113 (21.2%) had clinically meaningful worsening of LSM (≥30% increase). Overall, 89/113 patients (78.8%) had stable or improved liver stiffness at Month 36 of seladelpar treatment (<30% worsening or any improvement). Among the 19 patients with clinically meaningful LSM improvement at Month 36, 57.9% (11/19) began as low risk and remained low risk; 15.8% (3/19) began as intermediate risk and improved to low risk, and 26.3% (5/19) began as high risk and improved to intermediate risk (2/5; 40.0%) or low risk (3/5; 60.0%), as shown in Fig. 3A. Among the 24 patients with clinically meaningful LSM worsening, most (19/24; 79.2%) were low risk at baseline. At Month 36, over half (10/19; 52.6%) of these patients remained low risk, while 8/19 (42.1%) progressed to intermediate risk and 1/19 (5.3%) progressed to high risk. The remaining 5 patients with worsening were intermediate or high risk at baseline, and all were at high risk at Month 36 (Fig. 3B). Baseline and on-treatment characteristics for the 6 patients who experienced clinically meaningful worsening and were at high risk at Month 36 are described in Table S4.

Factors associated with LSM stability/improvement at Month 36

In a univariate analysis of the 113 patients with LSM data at Month 36, several factors were associated with stable or improved LSM (<30% worsening or any improvement) *vs.*

Table 1. Baseline demographic and clinical characteristics in the overall population and by baseline LSM risk category. Q9

	Overall (N = 307)	Low risk: Baseline LSM <10.7 kPa* (n = 223)	Intermediate risk: Baseline LSM ≥10.7 to <16.9 kPa* (n = 51)	High risk: Baseline LSM ≥16.9 kPa* (n = 33)
Age (years), mean (SD)	58.5 (9.6)	58.5 (9.3)	57.6 (11.7)	59.8 (8.1)
Sex, n (%)				
Female	290 (94.5)	213 (95.5)	50 (98.0)	27 (81.8)
Male	17 (5.5)	10 (4.5)	1 (2.0)	6 (18.2)
BMI ≥30 (kg/m^2), n (%)	85 (27.7)	56 (25.1)	19 (37.3)	10 (30.3)
ALP (U/L), mean (SD)**	288.0 (128.5)	282.5 (119.4)	307.2 (168.6)	295.2 (116.8)
Total bilirubin (mg/dl), mean (SD)	0.7 (0.3)	0.7 (0.3)	0.9 (0.4)	1.0 (0.4)
ALT (U/L), mean (SD)†	42.8 (23.4)	41.2 (23.3)	49.9 (25.0)	43.2 (20.3)
AST (U/L), mean (SD)‡	37.9 (16.5)	35.4 (15.6)	44.7 (17.3)	43.9 (17.2)
GGT (U/L), mean (SD)ˆ	227.9 (198.0)	211.4 (197.4)	269.5 (159.9)	274.9 (240.0)
ALB (g/dl), mean (SD)	4.1 (0.2)	4.2 (0.3)	4.1 (0.3)	4.0 (0.3)
Platelet (10^3/µl), mean (SD)	240.4 (79.0)	255.5 (74.3)	201.0 (82.1)	200.9 (72.3)
INR, mean (SD)	1.0 (0.1)	1.0 (0.1)	1.0 (0.1)	1.0 (0.1)
LSM (kPa), mean (SD)	9.8 (7.4)	6.8 (1.8)	13.0 (1.7)	25.8 (12.2)
ELF score, mean (SD)	10.0 (1.0)	9.7 (0.9)	10.7 (0.9)	11.0 (1.2)
Cirrhosis: n (%)§	49 (16.0)	9 (4.0)	14 (27.5)	26 (78.8)
Child-Pugh-A, n (%)¶	46 (93.9)	9 (100)	14 (100)	23 (88.5)
Child-Pugh-B, n (%)¶	3 (6.1)	0	0	3 (11.5)
Child-Pugh score, mean (SD)¶	5.2 (0.5)	5.1 (0.3)	5.1 (0.3)	5.3 (0.7)

ALB, albumin; ALP, alkaline phosphatase; ALT, alanine aminotransferase; AST, aspartate aminotransferase; ELF, enhanced liver fibrosis; GGT, gamma-glutamyltransferase; INR, international normalized ratio; LSM, liver stiffness measurement.

*Baseline was defined as the time of seladelpar initiation (at entry to RESPONSE for patients randomized to seladelpar in RESPONSE, and at entry to ASSURE for all other patients). LSM thresholds were used to group patients by baseline LSM into the following three risk categories: low (LSM <10.7 kPa), intermediate (LSM ≥10.7 to <16.9 kPa), and high (LSM ≥16.9 kPa).

**The ULN for ALP is 116 U/L in men and women.

†The ULN for ALT is 41 U/L in men and women.

‡The ULN for AST is 34 U/L in men and women.

ˆThe ULN for GGT is 52 U/L in men and 38 U/L in women.

§Cirrhosis at baseline was defined per-protocol and diagnostic criteria included liver biopsy, clinical history, laboratory findings, imaging findings, and/or clinical determination by the investigator. Of the 49 patients with protocol-defined cirrhosis, 23 (46.9%) had baseline LSM <16.9 kPa. Full cirrhosis diagnosis criteria have been reported previously.[29]

¶Among patients with cirrhosis.

clinically meaningful worsening (≥30%) from baseline (Table 2). Compared with patients who experienced clinically meaningful worsening at Month 36 (n = 24), those with stable or improved LSM (n = 89) were older at baseline (mean age of 60.1 *vs.* 55.7 years; $p = 0.0441$) and more frequently had baseline ALP <1.5× ULN (19.1% *vs.* 0%; $p = 0.0207$). Percentage change from baseline in ALP at Month 12 was not correlated with percentage change from baseline in LSM at Month 36 (Pearson's correlation coefficient: 0.095). However, achievement of CBR at Month 12 was significantly associated with LSM stability or improvement ($p = 0.0021$), as was achievement of its individual components and achievement of ALP <1.5× ULN at Month 12 ($p = 0.0193$; Table 2). Analyses using thresholds for LSM worsening of ≥20% and >0% yielded similar results (Table S5 and S7).

In a multivariate logistic regression model that included variables reaching significance in the univariate analysis and used stepwise variable selection, achievement of CBR at Month 12 predicted LSM stability or improvement with an OR (95% CI) of 5.13 (1.78, 14.76; $p = 0.0024$). Changes in LSM were further examined by degree of biochemical response. Patients who achieved ALP normalization or CBR with ALP >ULN were more likely to have stable or improved LSM (84.0% and 83.7%, respectively) compared with patients who did not achieve CBR at Month 36 (55.0%; Table 3). Logistic regression showed greater odds of LSM stability or improvement among patients who achieved a biochemical response (OR [95% CI]: 4.30 [1.35, 13.71] for ALP normalization and 4.21 [1.27, 13.92] for CBR with ALP >ULN; Table 3).

Additional analyses demonstrated that the odds of stability or improvement, compared with ≥20% and >0% increases in LSM, were similarly higher for patients who achieved biochemical response. Using these thresholds, the odds of stability or improvement were highest for patients who achieved ALP normalization (OR [95% CI]: 6.58 [2.11, 20.52] and 6.00 [1.75, 20.59] for <20% and ≤0%, respectively), followed by those who achieved CBR with ALP >ULN (OR [95% CI]: 5.40 [1.72, 17.00] and 5.56 [1.59, 19.43], respectively, compared with non-responders; Table S6 and S8).

Discussion

These interim analyses from the open-label ASSURE study provide valuable long-term data on the impact of seladelpar on liver stiffness, a measure that helps predict the risk of liver transplantation and death. Literature suggests LSM tends to increase over time in patients with PBC who have significant fibrosis or cirrhosis, with a threshold of 10 kPa indicating risk of LSM worsening and adverse outcomes.[2] In this ongoing study, mean liver stiffness remained stable with seladelpar through up to 3 years of treatment. Among the subset of over 100 patients who had reached 3 years on study, the majority (78.8%) demonstrated stable or improved liver stiffness with seladelpar treatment, with improvement defined as a decline of ≥30% from baseline. Patients in ASSURE with baseline LSM ≥10.7 to <16.9 kPa had stable LSM over time. Among those at the highest risk of progression based on LSM ≥16.9 kPa, liver stiffness was reduced over the treatment period; among the 11

UNCORRECTED PROOF

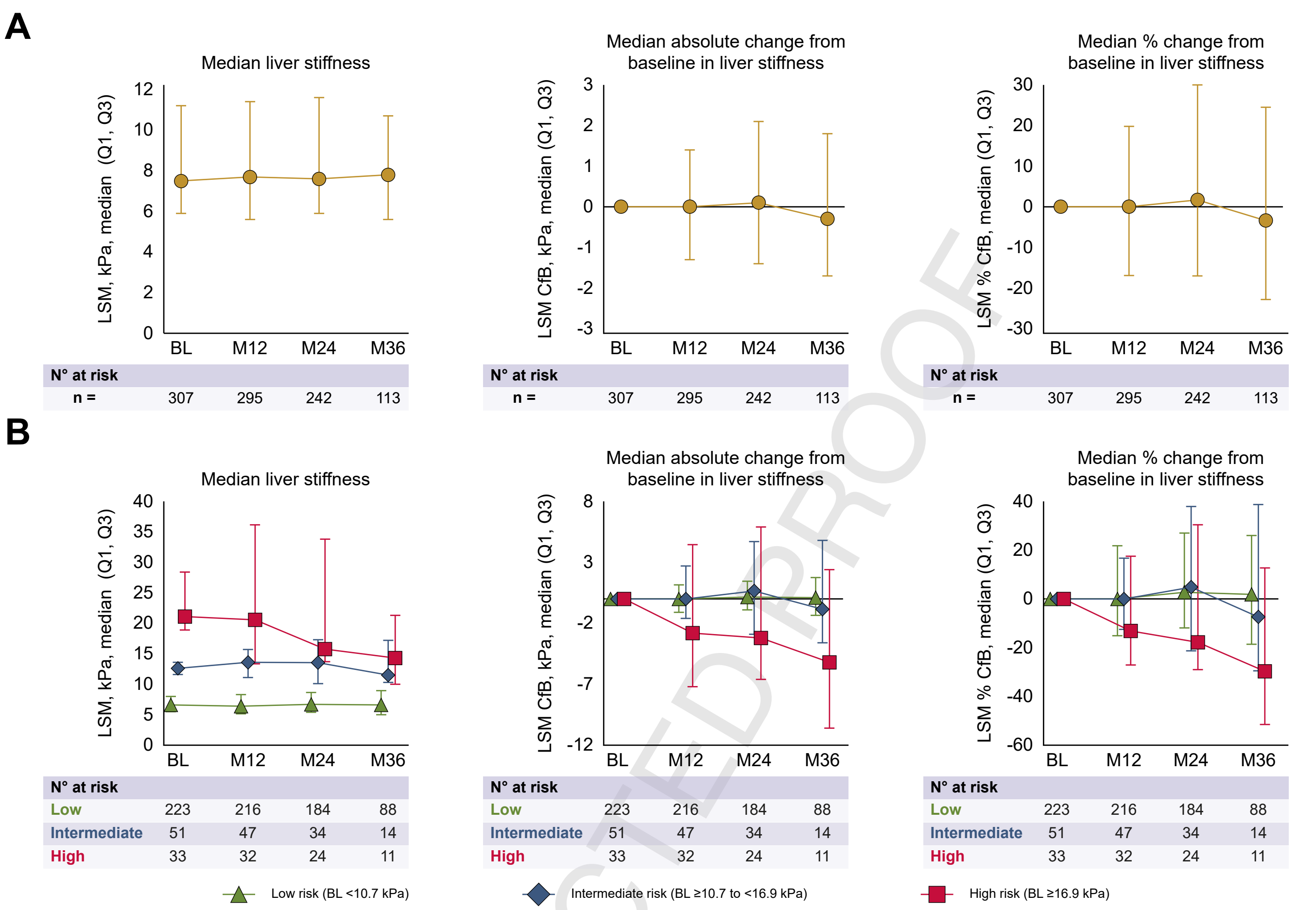

Fig. 1. Median LSM to Month 36. (A) Median LSM in the overall population. (B) Median LSM by baseline LSM risk category. BL, baseline; CfB, change from baseline; LSM, liver stiffness measurement; M, month. Q2

patients who had reached 3 years of treatment as of the analysis data cut-off, median percentage change in LSM was –29.7%.

Approximately one-fifth of the patients evaluable at Month 36 (n = 24) experienced a ≥30% increase in LSM. Of these patients, 10 were low risk at baseline (LSM <10.7 kPa) and remained low risk at 3 years, whereas 8 patients moved to intermediate risk (LSM ≥10.7 to <16.9 kPa), 5 moved to high risk (LSM ≥16.9 kPa), and 1 started at and remained high risk. In light of recent data showing that a patient's most recent LSM is the strongest predictor of hepatic decompensation in PBC, the risk of disease progression may remain low for patients who maintained LSM <10.7 kPa despite experiencing increases in LSM.[11]

To better characterize the patients in this study who experienced clinically meaningful LSM worsening, associations between their baseline characteristics and LSM trends were carefully evaluated. Patients with LSM worsening tended to be younger at baseline and had higher baseline ALP levels, with baseline ALP ≥1.5× ULN emerging as a significant risk factor. Patients with LSM worsening at 3 years were also significantly less likely to have met biochemical response

	Month 36 risk category,* n/N (%) (n = 113)		
Baseline risk category*	Low risk	Intermediate risk	High risk
Low risk (n = 88)	76/88 (86.4)	11/88 (12.5)	1/88 (1.1)
Intermediate risk (n = 14)	5/14 (35.7)	5/14 (35.7)	4/14 (28.6)
High risk (n = 11)	3/11 (27.3)	4/11 (36.4)	4/11 (36.4)

Shift to lower-risk category | Stable risk category | Shift to higher-risk category

Fig. 2. Shift table of change in LSM risk category from baseline to Month 36. *Patients were grouped by baseline LSM (kPa) into low (<10.7), intermediate (≥10.7 to <16.9), and high (≥16.9) risk. LSM, liver stiffness measurement, PBC, primary biliary cholangitis.

UNCORRECTED PROOF

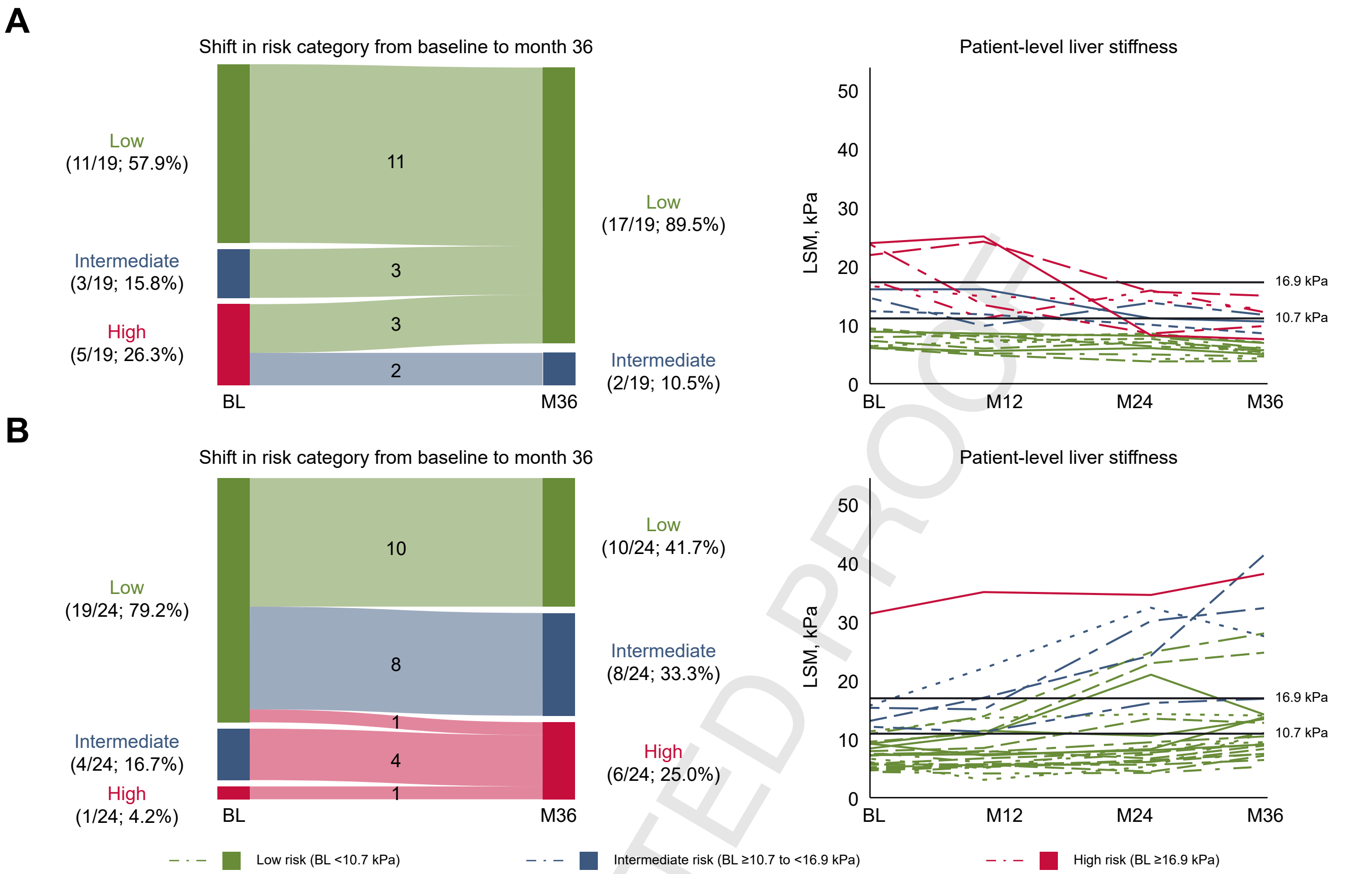

Fig. 3. Shifts from baseline and individual LSM values over time in patients with clinically meaningful (≥30%) change in LSM. (A) Patients with clinically meaningful improvement. (B) Patients with clinically meaningful worsening. BL, baseline; LSM, liver stiffness measurement; M, month.

criteria at Month 12. The univariate analysis demonstrated that achievement of various response thresholds, including CBR, was significantly associated with LSM stability or improvement. Achievement of ALP normalization did not reach significance in the univariate analysis, likely due to reduced power from the smaller number of patients. However, when patients who achieved ALP normalization or ALP >ULN but <1.67× ULN were compared with those who had ALP ≥1.67× ULN, ALP normalization was associated with the highest odds of LSM stability or improvement across multiple thresholds of LSM worsening.

Established risk factors for disease progression in PBC, including younger age and higher ALP, were more common among patients who experienced clinically meaningful LSM worsening in this study. Previous studies have similarly shown that elevated ALP levels, reflecting active cholestasis, are associated with increases in LSM over time.[23] These findings align with existing evidence that younger age and elevated ALP confer greater risk of adverse clinical outcomes[24,25] and with recent studies highlighting the importance of ALP reduction in lowering risk of disease progression.[6,7]

The association between on-treatment biochemical response and favorable liver stiffness outcomes observed here is also consistent with studies of earlier PBC therapies. In studies of obeticholic acid and off-label bezafibrate, patients who did not receive or did not adequately respond to treatment generally experienced LSM worsening, whereas those who achieved biochemical response based on ALP demonstrated more favorable LSM trajectories.[23,26]

The interplay between cholestasis reduction and potential improvement in fibrosis in PBC as assessed by VCTE remains an open question. Transient elastography measures shear-wave velocity to estimate liver stiffness, and is influenced by fibrosis, inflammation, and cholestasis, in addition to other factors.[9,10] Evidence of portal hypertension may also be present in patients with newly diagnosed PBC or elevated ALP in the absence of advanced fibrosis by LSM, suggesting a complex interplay between LSM, cholestasis, fibrosis, and the clinical manifestations of portal hypertension in PBC.[27] However, LSM has been shown to correlate with fibrosis stage in PBC, as well as other diseases, and is an independent predictor of clinical outcomes in PBC.[8–10] The time course of treatment effects on cholestatic markers and LSM, along with the lack of direct correlation between percentage change in ALP and LSM change, also suggests these assessments provide distinct clinical information, as seen when comparing the rapid effects of seladelpar on cholestatic markers (by 1 month), with LSM tending to remain stable or gradually decline. Overall, evidence indicates that LSM provides distinct prognostic information in patients with PBC receiving first-line or second-line treatment. These observations are reflected in a recent EASL-AASLD recommendation for LSM as a non-invasive measure of fibrosis to be considered as a surrogate endpoint in PBC clinical trials.[28] On this basis, the data Q4

UNCORRECTED PROOF

ARTICLE IN PRESS

Table 2. Univariate analysis for factors associated with LSM stability or improvement *vs.* ≥30% worsening from baseline at Month 36.

	Stability/improvement (<30% worsening or any improvement) (n = 89)	Clinically meaningful worsening (≥30% increase) (n = 24)	*p* value
**Baseline characteristics**			
Age (years), mean (SD)	60.1 (9.2)	55.7 (9.1)	0.0441*
Age at PBC diagnosis (years), mean (SD)	49 (8.1)	46 (7.6)	0.1728
Female, n (%)	84 (94.4)	22 (91.7)	0.6259
BMI ≥30 (kg/m^2), n (%)	32 (36.0)	6 (25.0)	0.3155
LSM (kPa), mean (SD)**	9.1 (5.1)	8.7 (5.4)	0.7335
≥10 kPa, n (%)	24 (27.0)	7 (29.2)	0.8310
ELF, mean (SD)	9.7 (0.8)	9.7 (1.0)	0.9161
AST (U/L), mean (SD)	34.2 (11.8)	38.3 (16.1)	0.2554
ALT (U/L), mean (SD)	39.4 (18.1)	48.8 (27.0)	0.1613
ALP (U/L), mean (SD)	260.2 (91.2)	293.6 (112.9)	0.2828
<1.5× ULN, n (%)	17 (19.1)	0	0.0207*
Total bilirubin (mg/dl), mean (SD)†	0.7 (0.2)	0.8 (0.3)	0.1233
GGT (U/L), mean (SD)	205.6 (206.4)	240.8 (165.6)	0.1521
ALB (g/dl), mean (SD)	4.2 (0.3)	4.2 (0.3)	0.9720
Platelet (10^3/µl), mean (SD)	243.3 (76.3)‡	235.0 (61.4)	0.4738
Cirrhosis, n (%)	14 (15.7)	2 (8.3)	0.3584
**On-treatment biochemical response at Month 12**			
CBR, n (%)	77 (86.5)	14 (58.3)	0.0021*
ALP <1.67× ULN, n (%)	79 (88.8)	17 (70.8)	0.0299*
ALP ≥15% reduction, n (%)	88 (98.9)	20 (83.3)	0.0011*
Total bilirubin ≤ULN, n (%)	87 (97.8)	21 (87.5)	0.0309*
ALP <1.5× ULN, n (%)	75 (84.3)	15 (62.5)	0.0193*
ALP ≤ULN, n (%)	42 (47.2)	8 (33.3)	0.2272

Statistical differences were assessed using a two-sided Wilcoxon rank-sum test for continuous variables and a Cochran-Mantel-Haenszel test for general association for categorical variables, performed for each factor in each comparison.
ALB, albumin; ALP, alkaline phosphatase; ALT, alanine aminotransferase; AST, aspartate aminotransferase; CBR, composite biochemical response; ELF, Enhanced Liver Fibrosis; GGT, gamma-glutamyltransferase; LSM, liver stiffness measurement; PBC, primary biliary cholangitis; ULN, upper limit of normal.
*$p < 0.05$.
**Baseline LSM (kPa) thresholds evaluated for statistical significance in the univariate analysis included: <10.7, ≥16.9, and an among-group comparison of <10.7 *vs.* ≥10.7 to <16.9 *vs.* ≥16.9; none reached significance at a level of $p < 0.05$.
†Total bilirubin ≤ULN and <0.6× ULN were additionally analyzed and did not reach significance.
‡Assessed in n = 87 patients.

presented here reflect the potential for seladelpar to stabilize or improve fibrosis over time in patients with PBC. However, the mechanism by which PBC treatments improve LSM and may improve fibrosis merits further evaluation. Whether different second-line treatments provide distinct benefits for liver stiffness, or fibrosis directly, also remains uncertain and may require longer-term follow up.

These analyses used a threshold of ≥30% change from baseline, either worsening or improvement, as prior studies across compensated advanced chronic liver disease, steatotic liver disease, and early real-world evidence in PBC suggest that a 20–30% change from baseline in LSM is clinically meaningful.[10,21,22] An analysis of the empirical distribution of percentage change in LSM at Month 36 in this cohort showed that changes of –30% and +30% approximately corresponded to the 20th and 80th percentiles of the distribution, respectively. Additional sensitivity analyses using thresholds of ≥20% change from baseline and >0% (any change) from baseline consistently showed that biochemical response was associated with LSM stability over the study period. Moreover, LSM trends over time were similar whether patients were grouped by the thresholds outlined in Corpechot *et al.* (2012) or those from the Baveno VII workshop.[9,21] Analyses of shifts between LSM risk categories were also overall similar in terms of proportion of patients having stable or improved LSM. Thresholds for clinically meaningful LSM change in PBC warrant further exploration.

Table 3. Odds of LSM stability or improvement *vs.* ≥30% worsening at Month 36 by biochemical response at Month 12.

	n	Stability/improvement (<30% worsening or any improvement) (n = 89)	Clinically meaningful worsening (≥30% increase) (n = 24)
Biochemical response at Month 12, n (%)			
ALP normalization	50	42 (84.0)	8 (16.0)
CBR with ALP >ULN	43	36 (83.7)	7 (16.3)
CBR non-response (reference)*	20	11 (55.0)	9 (45.0)
Odds ratio for LSM stability/improvement** at Month 36 (95% CI)			
ALP normalization†	–	4.30 (1.35, 13.71)	
CBR with ALP >ULN†	–	4.21 (1.27, 13.92)	

A logistic regression model with achievement of biochemical response at Month 12 as the fixed effect was used for comparison.
ALP, alkaline phosphatase; CBR, composite biochemical response; LSM, liver stiffness measurement; ULN, upper limit of normal.
*This group included patients who did not achieve CBR or ALP normalization.
**Stability/improvement defined as <30% worsening or any improvement in LSM.
†Compared with CBR non-response reference group.

UNCORRECTED

Strengths of these analyses include the evaluation of a large, well-characterized, prospective clinical study population of over 300 patients with up to 3 years of follow-up and over 100 patients who had reached 3 years of exposure in this ongoing study. In addition, subgroup analyses offered insight into LSM trajectories across patients with varying risk of disease progression based on baseline LSM.

Limitations include the open-label study design, absence of placebo control, and exploratory nature of the analyses, which restrict the ability to draw causal conclusions from these data. In the absence of a placebo control, further validation that the observed LSM trajectories do not reflect regression to the mean or within-patient variability, particularly in analyses stratified by baseline risk category, was not possible. The lack of centralized LSM readings prevented systematic quality control, and measurements with an IQR ≥30% were manually excluded rather than re-tested. Additionally, sample sizes at later timepoints in this study were limited, particularly within the high-risk subgroup of patients with baseline LSM ≥16.9 kPa. This raises the question of selection based on attrition; however, a pattern of improvement was observed at all timepoints among patients in this group, including earlier timepoints with higher patient numbers, supporting the findings observed at later timepoints. Small sample sizes may have also reduced the ability to detect statistical differences between associations of ALP and composite biochemical response with changes in LSM. Indeed, the wider LSM IQRs at later time points likely reflect both the smaller sample size available at those visits and the intrinsic variability of LSM by VCTE. Lastly, long-term clinical outcomes were not available for this interim analysis, and determining whether the favorable LSM trends observed here correspond to improved transplant-free survival will require long-term follow-up and additional analyses, including real-world evidence.

In this interim analysis of liver stiffness data from the open-label ASSURE study, mean liver stiffness in the overall population remained stable. Among patients who had reached 3 years on study, the majority demonstrated stable or improved LSM with seladelpar treatment. Notably, reductions in liver stiffness were observed in patients with the highest baseline liver stiffness values, a subgroup in which liver stiffness typically worsens despite UDCA treatment. Achievement of biochemical response with seladelpar at 1 year was associated with greater odds of maintaining or improving liver stiffness measures through 3 years of follow-up. Overall, this evidence supports the potential for seladelpar to favorably influence the disease course of PBC. It further supports the importance of ALP reduction in lowering the risk of disease progression based on non-invasive measures of fibrosis, although mechanistic questions remain about the relationship between changes in liver stiffness and cholestasis. These observations are important for patients with PBC and their healthcare providers considering the use of second-line therapy and individualized treatment goals. Long-term implications of these findings, particularly regarding PBC clinical outcomes, will require confirmation in future analyses.

Affiliations

[1]*Division of Gastroenterology and Hepatology, University of California Davis School of Medicine, Sacramento, CA, USA;* [2]*The Autoimmune and Rare Liver Disease Programme, Toronto General Hospital, Toronto, ON, Canada;* [3]*The Liver Autoimmunity Unit, Hospital Italiano de Buenos Aires, Buenos Aires, Argentina;* [4]*Liver Institute Northwest, Seattle, WA, USA;* [5]*Department of Internal Medicine, Saint Louis University School of Medicine, St. Louis, MO, USA;* [6]*Reference Center for Inflammatory Biliary Diseases and Autoimmune Hepatitis, French Network for Rare Liver Disease in Children and Adults (FILFOIE), European Reference Network RARE-LIVER, Saint-Antoine Hospital and Research Center, Assistance Publique-Hôpitaux de Paris, Sorbonne University, Paris, France;* [7]*Department of Gastroenterology and Hepatology, University Hospital Zürich, University of Zürich, Zürich, Switzerland;* [8]*National Institute for Health and Care Research Birmingham Biomedical Research Centre, Centre for Liver and Gastrointestinal Research, College of Medical and Dental Sciences, University of Birmingham, Birmingham, UK;* [9]*Liver Unit, University Hospitals Birmingham Queen Elizabeth, Birmingham, UK;* [10]*Barts Liver Centre, Blizard Institute, Queen Mary University of London, London, UK;* [11]*Department of Internal Medicine, Seoul National University Bundang Hospital, Seoul National University, College of Medicine, Seongnam, South Korea;* [12]*Gilead Sciences, Inc., Foster City, CA, USA;* [13]*Echosens, Paris, France;* [14]*Division of Digestive Health and Liver Diseases, Miller School of Medicine, University of Miami, Miami, FL, USA*

Abbreviations

ALP, alkaline phosphatase; CBR, composite biochemical response; LSM, liver stiffness measurement; OR, odds ratio; PBC, primary biliary cholangitis; UDCA, ursodeoxycholic acid; ULN, upper limit of normal; VCTE, vibration-controlled transient elastography.

Financial support

Gilead Sciences, Inc.

Conflict of interest

CLB reports receiving grants or contracts to his institution from Boston Scientific; Bristol Myers Squibb; Calliditas Therapeutics; Cara Therapeutics; Chemomab Therapeutics; COUR Pharmaceuticals; CymaBay Therapeutics; Gilead Sciences, Inc.; GSK; and Hanmi Pharmaceuticals; and consulting fees from Alnylam Pharmaceuticals; Chemomab Therapeutics; CymaBay Therapeutics; Gilead Sciences, Inc.; GSK; Ipsen; and NGM Bio. GMH reports having consulted for Advanz Pharma; Chemomab; CymaBay Therapeutics; Falk; Gilead Sciences, Inc.; GSK; Intercept Pharmaceuticals; Ipsen; Kezar; Kowa; Mirum Pharma; and Pliant Therapeutics. AMV reports receiving speaker fees from Intercept Pharmaceuticals and participation on an advisory board with Novartis. KVK reports receiving grants or contracts from 89Bio, Altimmune, Akero Therapeutics, Boehringer Ingelheim, Boston Pharmaceuticals, Corcept Therapeutics, Gilead Sciences, Inc, GlaxoSmithKline, Hanmi Pharmaceuticals, HighTide Biopharma, Ipsen, Inventiva, Kowa, Madrigal Pharmaceuticals, Mirum Pharma, and Zydus Pharmaceuticals; royalties or licenses from UpToDate; consulting fees from 89Bio, Akero Therapeutics, Boehringer Ingelheim, Corcept Therapeutics, Gilead Sciences, Inc., GlaxoSmithKline, Inipharm, Ipsen, Madrigal Pharmaceuticals, Mirum Pharma, Novo Nordisk, and Zydus Pharmaceuticals; payment or honoraria for lectures, presentations, speakers bureaus, manuscript writing, or educational events from Gilead Sciences, Inc., Ipsen, Madrigal Pharmaceuticals, and Novo Nordisk; and stock or stock options from Inipharm. HE reports receiving grants or honoraria from AbbVie; CymaBay Therapeutics; Eisai; Fibronostics; Genfit; Gilead Sciences, Inc.; Intercept Pharmaceuticals; and Mallinckrodt Pharmaceuticals; and owns equity in AbbVie and Gilead Sciences, Inc. CC reports consulting for Advanz; Calliditas; CymaBay Therapeutics; Falk Pharma; Gilead Sciences, Inc.; GSK; Intercept Pharmaceuticals; Ipsen; and Mirum Pharma; lecture fees from Echosens; and receiving research grants from Arrow; Intercept Pharmaceuticals; and Siemens Healthineers. AEK reports receiving grants or contracts from Gilead Sciences, Inc.; and Roche; consulting fees from AbbVie; Advanz Pharma; Alentis Therapeutics; Alfasigma; Astellas Pharma; AstraZeneca; Attovia Therapeutics; Avior Bio; Bayer; Bristol Myers Squibb; Böehringer Ingelheim; Curatis; Dr. Falk Pharma; Gilead Sciences, Inc.; GSK; Guidepoint; Ipsen; Merck Sharp & Dohme; Mirum Pharma; Novo Nordisk; Roche; and Takeda; payment or honoraria for lectures, presentations, speakers bureaus, manuscript writing, or educational events from AbbVie; Advanz Pharma; Bristol Myers Squibb; Falk Foundation; Gilead Sciences, Inc.; GSK;

UNCORRECTED PROOF

Ipsen; Johnson & Johnson; Medscape; Merck Sharp & Dohme; Mirum Pharma; Novartis; and Roche; support for attending meetings and/or travel from Gilead Sciences, Inc.; and a leadership or fiduciary role in other board, society, committee, or advocacy groups (paid or unpaid) with PBC Foundation, Swiss Association for the Study of the Liver (SASL), Swiss Gastroenterology Society (SGG), Swiss Hepa, and Swiss Transplant Society (STS). PJT reports receiving institutional funding support from the NIHR; lecture fees from Advanz Pharma/Intercept Pharmaceuticals, Albireo/Ipsen, CymaBay Therapeutics, and Dr. Falk Pharma; consulting fees from Advanz Pharma/Intercept Pharmaceuticals; Albireo/Ipsen; Chemomab Therapeutics; CymaBay Therapeutics/Gilead Sciences, Inc.; Dr. Falk Pharma; Mirum Pharma; and Pliant Therapeutics; and grant support from Advanz Pharma/Intercept Pharmaceuticals; Albireo/Ipsen; Bristol Myers Squibb; Core (Guts UK); European Association for the Study of the Liver; Gilead Sciences, Inc.; GSK; Innovate UK; Ipsen; LifeArc; Mirum Pharma; NIHR; PSC Support; Regeneron Pharmaceuticals; The Wellcome Trust; and The Medical Research Foundation (UK). YK received consulting fees from Dr. Falk Pharma and Ipsen; received payment or honoraria for lectures, presentations, speakers bureaus, manuscript writing, or educational events from Advanz Pharma; Dr. Falk Pharma; Gilead Sciences, Inc.; and Ipsen; and received support for attending meetings and/or travel from Advanz Pharma; Dr. Falk Pharma; Gilead Sciences, Inc.; and Ipsen. SHJ reports receiving institutional fees for seladelpar clinical trial participation from CymaBay Therapeutics, a Gilead Sciences, Inc., company. XQ is an employee of Gilead Sciences, Inc., and may own stock in Gilead Sciences, Inc. KmK was an employee of Gilead Sciences, Inc. at the time this research was conducted, and may own stock in Gilead Sciences, Inc. DBC was an employee of CymaBay Therapeutics, a Gilead Sciences Company, at the time the work was conducted, and is an employee of Gilead Sciences, Inc. and may own stock in Gilead Sciences, Inc. VdL is an employee of Echosens. CL reports receiving research grants paid to her institution from Calliditas Therapeutics; CymaBay Therapeutics; Escient Pharmaceuticals; EWR; Gilead Sciences, Inc.; GSK; Intercept Pharmaceuticals; Ipsen; Kowa; Mirum Pharma; Target; and Zydus Pharmaceuticals.

Please refer to the accompanying ICMJE disclosure forms for further details.

Authors' contributions

Substantial contributions to study conception and design: CLB, CC, KmK, VdL, XQ, DBC, CL; substantial contributions to analysis and interpretation of the data: CLB, GMH, AMV, KVK, HE, CC, AEK, PJT, YK, SHJ, KmK, VdL, XQ, DBC, CL; drafting the article or reviewing it critically for important intellectual content: CLB, GMH, AMV, KVK, HE, CC, AEK, PJT, YK, SHJ, KmK, VdL, XQ, DBC, CL; final approval of the version of the article to be published: CLB, GMH, AMV, KVK, HE, CC, AEK, PJT, YK, SHJ, KmK, VdL, XQ, DBC, CL.

Data availability

Gilead Sciences shares anonymized individual patient data upon request or as required by law or regulation with qualified external researchers based on submitted curriculum vitae and reflecting non conflict of interest. The request proposal must also include a statistician. Approval of such requests is at Gilead Science's discretion and is dependent on the nature of the request, the merit of the research proposed, the availability of the data, and the intended use of the data. Data requests should be sent to datarequest@gilead.com.

Acknowledgements

The authors would like to thank all of the investigators, their clinical staff, and the patients who participated in the study. The authors also acknowledge Ari Navetta, BS, and Quinn Ho, PhD, of Costello Medical Inc., Boston, Massachusetts, USA, and Hannah Brechka, PhD, CMPP, of Costello Medical Inc., Cambridge, UK, for medical writing and editorial assistance based on the authors' input and direction. Support for third party medical writing was funded by Gilead Sciences, Inc.

Supplementary data

Supplementary data to this article can be found online at https://doi.org/10.1016/j.jhepr.2026.102057.

References

[1] Lleo A, Wang GQ, Gershwin ME, et al. Primary biliary cholangitis. Lancet 2020;396:1915–1926.
[2] European Association for the Study of the Liver. EASL Clinical Practice Guidelines: the diagnosis and management of patients with primary biliary cholangitis. J Hepatol 2017;67:145–172.
[3] Corpechot C, Chazouillères O, Poupon R. Early primary biliary cirrhosis: biochemical response to treatment and prediction of long-term outcome. J Hepatol 2011;55:1361–1367.
[4] Lammers WJ, van Buuren HR, Hirschfield GM, et al. Levels of alkaline phosphatase and bilirubin are surrogate end points of outcomes of patients with primary biliary cirrhosis: an international follow-up study. Gastroenterology 2014;147:1338–1349. e5; quiz e15.
[5] Kowdley KV, Ieyoub J, Pan Y, et al. Treatment Persistence, normal alkaline phosphatase and clinical outcomes in primary biliary cholangitis. Aliment Pharmacol Ther 2026;63:522–537.
[6] Corpechot C, Lemoinne S, Soret PA, et al. Adequate *vs.* deep response to ursodeoxycholic acid in primary biliary cholangitis: to what extent and under what conditions is normal alkaline phosphatase level associated with complication-free survival gain? Hepatology 2024;79:39–48.
[7] Murillo Perez CF, Harms MH, Lindor KD, et al. Goals of treatment for improved survival in primary biliary cholangitis: treatment Target should Be bilirubin within the normal Range and normalization of alkaline phosphatase. Am J Gastroenterol 2020;115:1066–1074.
[8] Corpechot C, Carrat F, Gaouar F, et al. Liver stiffness measurement by vibration-controlled transient elastography improves outcome prediction in primary biliary cholangitis. J Hepatol 2022;77:1545–1553.
[9] Corpechot C, Carrat F, Poujol-Robert A, et al. Noninvasive elastography-based assessment of liver fibrosis progression and prognosis in primary biliary cirrhosis. Hepatology 2012;56:198–208.
[10] Lam L, Soret PA, Lemoinne S, et al. Dynamics of liver stiffness measurement and clinical course of primary biliary cholangitis. Clin Gastroenterol Hepatol 2024;22:2432–2441.e2.
[11] Wong YJ, Lam L, Soret PA, et al. Prognostic value of liver stiffness measurement *vs.* biochemical response in primary biliary cholangitis. J Hepatol 2026;84:275–283.
[12] LIVDELZI (seladelpar). Prescribing information. Gilead Sciences. Foster City, California: Inc.; 2024. https://www.gilead.com/-/media/files/pdfs/medicines/pbc/livdelzi/livdelzi_pi.pdf. [Accessed 31 October 2025].
[13] LIVDELZI (seladelpar). Summary of Product characteristics. Gilead Sciences. Foster City, California: Inc.; 2025. https://www.ema.europa.eu/en/documents/product-information/seladelpar-gilead-epar-product-information_en.pdf. [Accessed 31 October 2025].
[14] LIVDELZI 10 mg capsules. Summary of Product characteristics. Gilead Sci Ltd : Lond UK 2025. https://mhraproducts4853.blob.core.windows.net/docs/62f9bb37d019209632ae2467559e90975e9edd92. [Accessed 11 February 2026].
[15] Hirschfield GM, Bowlus CL, Mayo MJ, et al. A phase 3 trial of seladelpar in primary biliary cholangitis. N Engl J Med 2024;390:783–794.
[16] Kouno T, Liu X, Zhao H, et al. Selective PPARδ agonist seladelpar suppresses bile acid synthesis by reducing hepatocyte CYP7A1 via the fibroblast growth factor 21 signaling pathway. J Biol Chem 2022;298:102056.
[17] Lim HJ, Kwak HJ. Selective PPARδ agonist GW501516 Protects against LPS-Induced Macrophage inflammation and Acute liver failure in Mice via Suppressing inflammatory Mediators. Molecules 2024;29:5189.
[18] Levy C, Trivedi PJ, Kowdley KV, et al. Long-term efficacy and safety of selective PPARδ agonist seladelpar in primary biliary cholangitis: ASSURE interim study results. Am J Gastroenterol 2025;121:1140–1153 [Epub ahead of print].
[19] Levy C, Trivedi PJ, Kowdley KV, et al. Long-term efficacy and safety of selective PPARδ agonist seladelpar in primary biliary cholangitis: ASSURE interim study results. Am J Gastroenterol 2025;(Suppl).
[20] International Council for Harmonisation of Technical Requirements for Pharmaceuticals for Human Use. Guideline for Good clinical Practice E6(R3). 2025. Available at: https://database.ich.org/sites/default/files/ICH_E6%28R3%29_Step4_FinalGuideline_2025_0106.pdf. [Accessed 30 October 2025].
[21] de Franchis R, Bosch J, Garcia-Tsao G, et al. Baveno VII - Renewing consensus in portal hypertension. J Hepatol 2022;76:959–974.
[22] Noureddin M, Charlton MR, Harrison SA, et al. Expert Panel recommendations: Practical clinical Applications for initiating and Monitoring Resmetirom in patients with MASH/NASH and moderate to Noncirrhotic advanced fibrosis. Clin Gastroenterol Hepatol 2024;22:2367–2377.
[23] Terracciani F, De Vincentis A, D'Amato D, et al. Long-term effectiveness, safety, and liver stiffness dynamics of PBC treatment with obeticholic acid in real-world. JHEP Rep 2025;7:101448.
[24] Cheung AC, Lammers WJ, Murillo Perez CF, et al. Effects of age and Sex of response to ursodeoxycholic acid and transplant-free survival in patients

UNCORRECTED PROOF

with primary biliary cholangitis. Clin Gastroenterol Hepatol 2019;17:2076–2084.e2.
[25] Murillo Perez CF, Hirschfield GM, Corpechot C, et al. Fibrosis stage is an independent predictor of outcome in primary biliary cholangitis despite biochemical treatment response. Aliment Pharmacol Ther 2019;50:1127–1136.
[26] Corpechot C, Chazouillères O, Rousseau A, et al. A placebo-controlled trial of bezafibrate in primary biliary cholangitis. N Engl J Med 2018;378:2171–2181.
[27] Calvaruso V, Celsa C, Cristoferi L, et al. Noninvasive assessment of portal hypertension in patients with primary biliary cholangitis is Affected by Severity of cholestasis. Clin Gastroenterol Hepatol 2025;23:1766–1775.e7.
[28] EASL-AASLD Delphi consensus statement on surrogate endpoints and real-world evidence in primary biliary cholangitis. J Hepatol 2026.
[29] Levy C, Trivedi PJ, Kowdley KV, et al. Long-term efficacy and safety of selective PPARδ agonist seladelpar in primary biliary cholangitis: ASSURE interim study results. Am J Gastroenterol 2025;121:1140–1153 [Suppl. Epub ahead of print].

Keywords: Primary biliary cholangitis; seladelpar; liver stiffness; FibroScan; non-invasive test; composite biochemical response.
Received 21 April 2026; received in revised form 21 September 2026; accepted 25 September 2026; Available online xxx

UNCORRECTED PROOF

Journal of Hepatology, Volume ■

Supplemental information

Seladelpar improves liver stiffness in PBC: ASSURE interim analysis

Christopher L. Bowlus, Gideon M. Hirschfield, Alejandra M. Villamil, Kris V. Kowdley, Hany Elbeshbeshy, Christophe Corpechot, Andreas E. Kremer, Palak J. Trivedi, Yiannis Kallis, Sook-Hyang Jeong, Kyung min Kwon, Victor de Ledinghen, Xin Qi, Daria B. Crittenden, Cynthia Levy, and on behalf of the ASSURE Investigators

Seladelpar improves liver stiffness in PBC: ASSURE interim analysis

Christopher L. Bowlus, Gideon M. Hirschfield, Alejandra M. Villamil, Kris V. Kowdley, Hany Elbeshbeshy, Christophe Corpechot, Andreas E. Kremer, Palak J. Trivedi, Yiannis Kallis, Sook-Hyang Jeong, Kyung min Kwon, Victor de Ledinghen, Xin Qi, Daria B. Crittenden, Cynthia Levy, on behalf of the ASSURE Investigators

Table of contents

Fig. S1.......... 2
Fig. S2.......... 4
Table S1.......... 5
Table S2.......... 6
Table S3.......... 7
Table S4.......... 8
Table S5.......... 9
Table S6.......... 10
Table S7.......... 11
Table S8.......... 12
Supplementary references.......... 13

SUPPLEMENTARY FIGURES

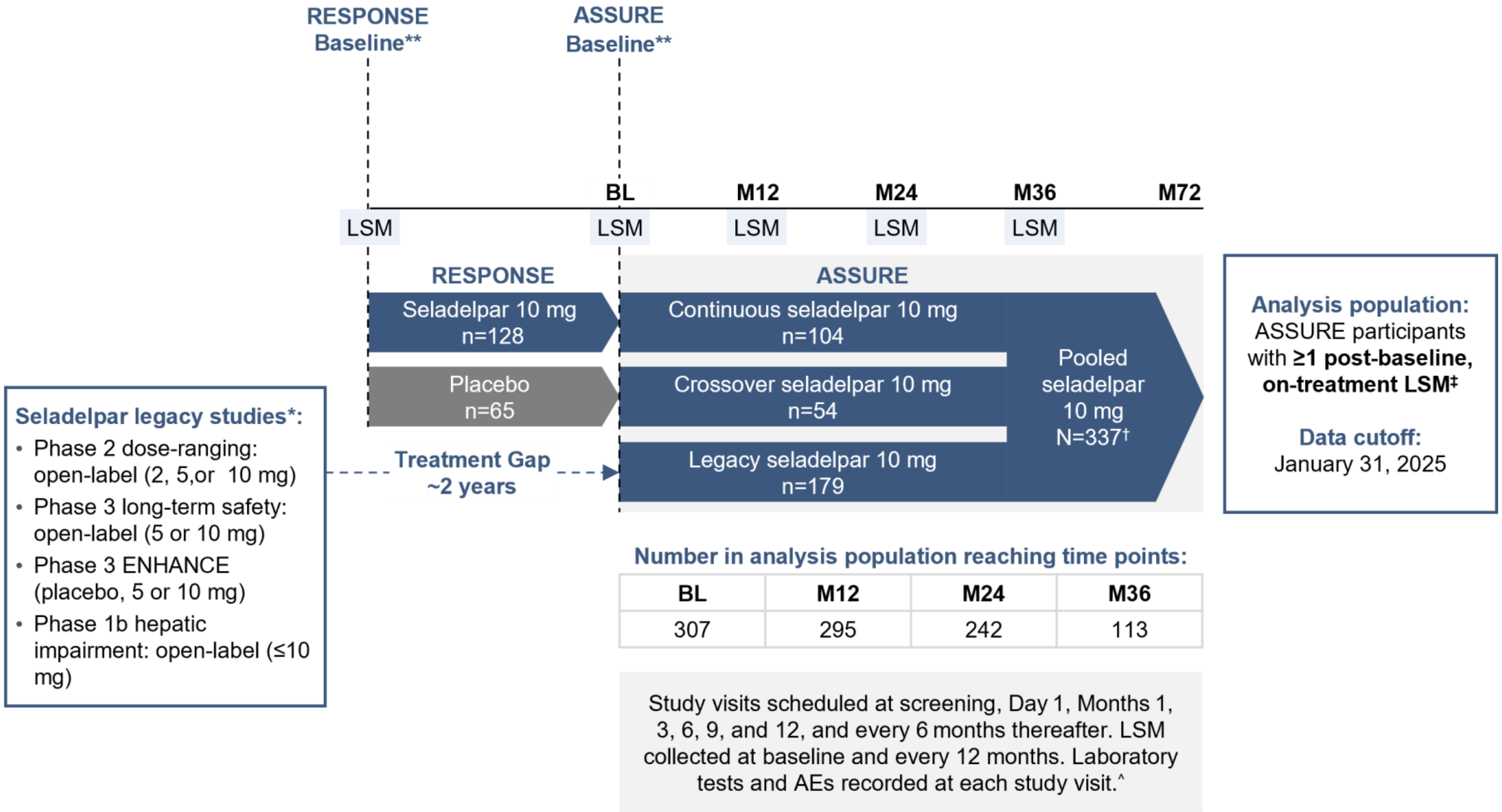

Fig. S1. ASSURE study schema.

*Legacy studies included the phase 2 dose-ranging study (NCT02955602),[1] phase 3 long-term safety study (NCT03301506), phase 3 ENHANCE study (NCT03602560),[2] and phase 1b hepatic impairment study (NCT04950764). The phase 3 placebo-controlled RESPONSE study (NCT04620733) and phase 3, open-label ASSURE study (NCT03301506) are registered at clinicaltrials.gov. The phase 2 and 3 parent studies required an inadequate response or intolerance to first-line UDCA; **For patients who enrolled in ASSURE from the seladelpar arm in RESPONSE, BL was defined as seladelpar initiation in RESPONSE and RESPONSE data were included in the analysis. For patients who crossed over from the placebo arm of RESPONSE or had previously participated in seladelpar legacy studies, baseline was defined as seladelpar initiation in ASSURE; †Treatment gaps between legacy studies and ASSURE ranged from 27 days (phase 1b impairment study) to 212 weeks (phase 3 ENHANCE study); ‡In ASSURE, 2 patients initiated seladelpar at 5 mg and were excluded from the analysis; ^As part of data quality procedures, LSM values with ≥50% increase or ≥30% decrease from the prior measurement were queried for accuracy of LSM and IQR. A reliable LSM was defined by an IQR/median ratio ≤30%, and measurements that did not meet this threshold were excluded from the analysis; §LSM was assessed in RESPONSE and ASSURE using VCTE and collected with local reads. Patients were instructed to fast for ≥4 hours prior to examination if possible. In RESPONSE, LSM was assessed at baseline and at Months 6 and 12; in ASSURE, LSM was assessed at screening and every 12 months thereafter. This analysis exclusively included LSMs collected using FibroScan® (Echosens, France). AE, adverse event; BL, baseline; IQR, interquartile range; LSM, liver stiffness measurement; M, month; UDCA, ursodeoxycholic acid; VCTE, vibration-controlled transient elastography.

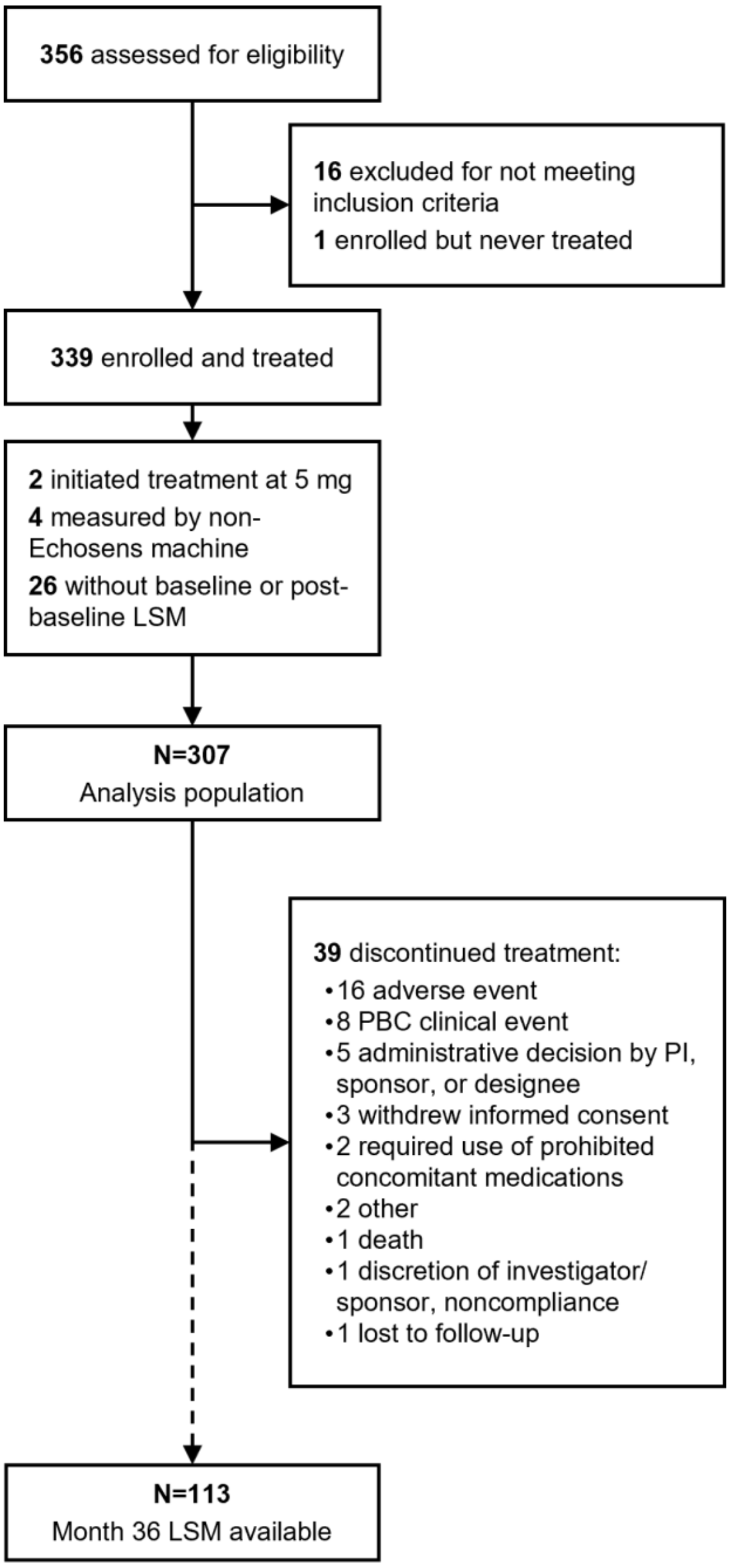

Fig. S2. ASSURE patient disposition for liver stiffness analysis population.

Interim analysis with data cutoff of January 31, 2025. In the analysis population, 145 patients rolled over to ASSURE from the phase 3 RESPONSE study and 162 enrolled from previous seladelpar legacy studies after meeting ASSURE eligibility criteria. *ASSURE is an ongoing study and some patients had not yet reached Month 36 prior to the data cut-off. LSM, liver stiffness measurement; PBC, primary biliary cholangitis; PI, principal investigator.

SUPPLEMENTARY TABLES

Table S1. Baseline demographic and clinical characteristics in the overall population and by baseline Baveno VII category.

	Overall (N=307)	Baseline LSM <10 kPa* (N=209)	Baseline LSM ≥10 to <15 kPa* (N=57)	Baseline LSM ≥15 kPa* (N=41)
**Age (years), mean (SD)**	58.5 (9.6)	58.4 (9.3)	57.6 (11.0)	59.9 (9.5)
**Sex, n (%)**				
**Female**	290 (94.5)	200 (95.7)	55 (96.5)	35 (85.4)
**Male**	17 (5.5)	9 (4.3)	2 (3.5)	6 (14.6)
**BMI ≥30 (kg/m^2), n (%)**	85 (27.7)	54 (25.8)	19 (33.3)	12 (29.3)
**ALP (U/L), mean (SD)****	288.0 (128.5)	279.2 (116.9)	308.4 (153.8)	304.5 (144.4)
**Total bilirubin (mg/dL), mean (SD)**	0.7 (0.3)	0.7 (0.3)	0.8 (0.3)	1.0 (0.4)
**ALT (U/L), mean (SD)†**	42.8 (23.4)	40.8 (23.2)	48.0 (25.4)	46.0 (20.6)
**AST (U/L), mean (SD)‡**	37.9 (16.5)	35.0 (15.7)	43.6 (17.4)	44.5 (15.9)
**GGT (U/L), mean (SD)^**	227.9 (198.0)	209.7 (200.4)	258.1 (160.6)	278.3 (222.0)
**ALB (g/dL), mean (SD)**	4.1 (0.3)	4.2 (0.3)	4.1 (0.3)	4.0 (0.3)
**Platelet (10^3/uL), mean (SD)**	240.4 (79.0)	256.4 (75.0)	209.2 (80.0)	202.2 (73.3)
**INR, mean (SD)**	1.0 (0.1)	1.0 (0.1)	1.0 (0.1)	1.0 (0.1)
**LSM (kPa), mean (SD)**	9.8 (7.4)	6.5 (1.6)	11.9 (1.3)	23.9 (11.6)
**ELF score, mean (SD)**	10.0 (1.0)	9.7 (0.8)	10.6 (0.8)	11.0 (1.1)
**Cirrhosis: n (%)§**	49 (16.0)	8 (3.8)	10 (17.5)	31 (75.6)
**CP-A, n (%)¶**	46 (93.9)	8 (100)	10 (100)	28 (90.3)
**CP-B, n (%)¶**	3 (6.1)	0	0	3 (9.7)
**Child-Pugh score, mean (SD)¶**	5.2 (0.5)	5.1 (0.4)	5.0 (0.0)	5.3 (0.6)

*Baseline was defined as the time of seladelpar initiation (at entry to RESPONSE for patients randomized to seladelpar in RESPONSE, and at entry to ASSURE for all other patients); **The ULN for ALP is 116 U/L in men and women; †The ULN for ALT is 41 U/L in men and women; ‡The ULN for AST is 34 U/L in men and women; ^The ULN for GGT is 52 U/L in men and 38 U/L in women; §Cirrhosis at baseline was defined per-protocol and diagnostic criteria included liver biopsy, clinical history, laboratory findings, imaging findings, and/or clinical determination by the investigator. Full cirrhosis diagnosis criteria have been reported previously[3]; ¶Among patients with cirrhosis. ALB, albumin; ALP, alkaline phosphatase; ALT, alanine aminotransferase; AST, aspartate aminotransferase; BMI, body mass index; CP-A, Child-Pugh class A; CP-B, Child-Pugh class B; ELF, Enhanced Liver Fibrosis; GGT, gamma-glutamyl transferase; INR, international normalized ratio; LSM, liver stiffness measurement.

Table S2. Median LSM over time. (A) Median LSM in the overall population. (B) Median LSM by baseline LSM risk category.

A

	Overall Population			
	**Baseline (N=307)**	**Month 12 (N=295)**	**Month 24 (N=242)**	**Month 36 (N=113)**
**LSM (kPa), median (Q1, Q3)**	7.5 (5.9, 11.2)	7.7 (5.6, 11.4)	7.6 (5.9, 11.6)	7.8 (5.6, 10.7)
**LSM (kPa), absolute change from BL, median (Q1, Q3)**	—	0 (−1.3, 1.4)	0.1 (−1.4, 2.1)	−0.3 (−1.7, 1.8)
**% LSM change from BL, median (Q1, Q3)**	—	0 (−16.9, 19.8)	1.7 (−17.0, 30.0)	−3.4 (−22.8, 24.5)

B

	Low Risk Baseline LSM <10.7 kPa				Intermediate Risk Baseline LSM ≥10.7 to <16.9 kPa				High Risk Baseline LSM ≥16.9 kPa			
	**Baseline (N=223)**	**Month 12 (N=216)**	**Month 24 (N=184)**	**Month 36 (N=88)**	**Baseline (N=51)**	**Month 12 (N=47)**	**Month 24 (N=34)**	**Month 36 (N=14)**	**Baseline (N=33)**	**Month 12 (N=32)**	**Month 24 (N=24)**	**Month 36 (N=11)**
**LSM (kPa) median (Q1, Q3)**	6.6 (5.5, 8.0)	6.4 (5.2, 8.3)	6.7 (5.4, 8.7)	6.6 (5.0, 9.0)	12.6 (11.6, 13.6)	13.6 (11.1, 15.7)	13.6 (10.1, 17.3)	11.5 (10.3, 17.2)	21.1 (18.9, 28.4)	20.6 (13.4, 36.2)	15.8 (13.7, 33.8)	14.3 (10.0, 21.3)
**LSM (kPa), absolute change from BL, median (Q1, Q3)**	—	0 (−1.1, 1.2)	0.2 (−0.9, 1.5)	0.1 (−1.4, 1.8)	—	0 (−1.6, 2.7)	0.7 (−2.9, 4.7)	−0.9 (−3.6, 4.8)	—	−2.8 (−7.2, 4.5)	−3.2 (−6.6, 5.9)	−5.2 (−10.6, 2.4)
**% LSM change from BL, median (Q1, Q3)**	—	0 (−15.1, 21.8)	2.7 (−11.9, 27.0)	1.8 (−18.6, 26.0)	—	0 (−12.5, 16.7)	4.8 (−21.3, 37.9)	−7.4 (−29.5, 38.7)	—	−13.1 (−27.1, 17.5)	−17.8 (−29.0, 30.4)	−29.7 (−51.5, 12.7)

An increase in liver stiffness from baseline ≥30% at any timepoint was considered clinically meaningful worsening, while a decrease in liver stiffness from baseline ≥30% was considered clinically meaningful improvement. BL, baseline; LSM, liver stiffness measurement; Q1, first quartile; Q3, third quartile.

Table S3. Median LSM over time by Baveno VII category.

	Baseline LSM <10 kPa				Baseline LSM ≥10 to <15 kPa				Baseline LSM ≥15 kPa			
	**Baseline (N=209)**	**Month 12 (N=202)**	**Month 24 (N=173)**	**Month 36 (N=82)**	**Baseline (N=57)**	**Month 12 (N=53)**	**Month 24 (N=39)**	**Month 36 (N=15)**	**Baseline (N=41)**	**Month 12 (N=40)**	**Month 24 (N=30)**	**Month 36 (N=16)**
**LSM (kPa) median (Q1, Q3)**	6.5 (5.4, 7.6)	6.2 (5.1, 8.0)	6.6 (5.4, 8.1)	6.4 (4.9, 8.3)	11.9 (10.8, 12.8)	12.0 (9.9, 14.4)	12.0 (9.6, 16.9)	11.2 (9.0, 16.3)	19.8 (17.5, 26.8)	16.4 (13.4, 27.2)	15.8 (13.7, 30.3)	14.5 (10.2, 21.2)
**LSM (kPa), absolute change from BL, median (Q1, Q3)**	—	0.0 (−1.0, 1.1)	0.1 (−0.9, 1.4)	0.1 (−1.4, 1.7)	—	0.2 (−1.5, 2.8)	0.9 (−2.6, 4.8)	−0.5 (−1.5, 4.8)	—	−1.9 (−7.1, 2.8)	−3.0 (−5.2, 5.5)	−3.9 (−6.2, 1.5)
**% LSM change from BL, median (Q1, Q3)**	—	0.0 (−15.9, 21.7)	2.0 (−11.8, 26.4)	1.6 (−19.2, 25.9)	—	1.5 (−12.5, 24.4)	8.7 (−20.5, 43.4)	−4.1 (−11.8, 38.7)	—	−9.7 (−27.1, 16.0)	−16.6 (−28.2, 24.8)	−22.0 (−37.3, 7.9)

BL, baseline; LSM, liver stiffness measurement; Q1, first quartile; Q3, third quartile.

Table S4. Characteristics of patients with ≥30% worsening and high-risk category at Month 36.

	Patient 1	Patient 2	Patient 3	Patient 4	Patient 5	Patient 6
**Baseline characteristics**						
**Age (years)**	46	55	49	63	66	55
**Age at PBC diagnosis (years)**	39	34	43	53	56	51
**Sex**	Female	Female	Female	Female	Female	Female
**ALP (U/L)***	223.0	349.5	230.0	183.0	324.3	647.0
**Total bilirubin (mg/dL)**	0.4	1.4	0.5	0.7	1.2	0.9
**LSM (kPa)**	8.7	15.4	12.6	12.4	29.8	11.1
**Cirrhosis****	No	No	Yes	No	Yes	No
**Child-Pugh, class (score)**	—	—	CP-A (5)	—	CP-A (5)	—
**On-treatment characteristics**						
**M12 CBR**	No	No	No	Yes	No	No
**M12 ALP ≤ULN**	No	No	No	Yes	No	No
**M36 ALP % change from baseline**	−11.7	−22.2	−37.0	−62.8	−40.8	−53.2
**M36 LSM (kPa)**	25.7	33.6	25.3	17.2	40.2	50.1

*The ULN for ALP is 116 U/L in men and women; **Cirrhosis at baseline was defined per-protocol and diagnostic criteria included liver biopsy, clinical history, laboratory findings, imaging findings, and/or clinical determination by the investigator. Full cirrhosis diagnosis criteria have been reported previously.[3] ALB, albumin; ALP, alkaline phosphatase; ALT, alanine aminotransferase; AST, aspartate aminotransferase; BMI, body mass index; CBR, composite biochemical response; CP-A, Child-Pugh class A; ELF, Enhanced Liver Fibrosis; GGT, gamma-glutamyl transferase; INR, international normalized ratio; LSM, liver stiffness measurement; M, month; PBC, primary biliary cholangitis; ULN, upper limit of normal.

Table S5. Univariate analysis for factors associated with LSM stability or improvement versus ≥20% worsening from baseline at Month 36.

	<20% worsening or any improvement (N=78)	≥20% Worsening (N=35)	p value
**Baseline characteristics**			
**Age (years), mean (SD)**	60.0 (9.5)	57.4 (8.8)	0.1876
**Age at PBC diagnosis (years), mean (SD)**	49 (8.3)	48 (7.8)	0.8133
**Female, n (%)**	73 (93.6)	33 (94.3)	0.8876
**BMI ≥30 (kg/m^2), n (%)**	30 (38.5)	8 (22.9)	0.1060
**LSM (kPa), mean (SD)****	9.3 (5.2)	8.3 (4.9)	0.4177
**≥10 kPa, n (%)**	23 (29.5)	8 (22.9)	0.4671
**ELF, mean (SD)**	9.7 (0.8)	9.7 (0.9)	0.7562
**AST (U/L), mean (SD)**	33.1 (11.4)	39.4 (14.9)	0.0225
**ALT (U/L), mean (SD)**	38.7 (18.0)	47.5 (24.5)	0.0832
**ALP (U/L), mean (SD)**	250.5 (81.5)	304.6 (116.8)	0.0353*
**<1.5× ULN, n (%)**	16 (20.5)	1 (2.9)	0.0157*
**Total bilirubin (mg/dL), mean (SD)**	0.7 (0.2)	0.8 (0.3)	0.0172*
**≤ULN, n (%)**	74 (94.9)	29 (82.9)	0.0384*
**<0.6× ULN, n (%)**	47 (60.3)	12 (34.3)	0.0110*
**GGT (U/L), mean (SD)**	204.4 (215.6)	232.3 (153.7)	0.0883
**ALB (g/dL), mean (SD)**	4.2 (0.3)	4.2 (0.3)	0.7325
**Platelet (10^3/uL), mean (SD)**	242.8 (80.9)†	238.6 (53.6)	0.8689
**Cirrhosis, n (%)**	13 (16.7)	3 (8.6)	0.2559
**On-treatment biochemical response at Month 12**			
**CBR, n (%)**	70 (89.7)	21 (60.0)	0.0002*
**ALP <1.67× ULN, n (%)**	72 (92.3)	24 (68.6)	0.0012*
**ALP ≥15% reduction, n (%)**	77 (98.7)	31 (88.6)	0.0158*
**Total bilirubin ≤ULN), n (%)**	76 (97.4)	32 (91.4)	0.1529
**ALP <1.5× ULN, n (%)**	68 (87.2)	22 (62.9)	0.0031*
**ALP ≤ULN, n (%)**	39 (50.0)	11 (31.4)	0.0673

Statistical differences were assessed using a two-sided Wilcoxon rank sum test for continuous variables and a Cochran-Mantel-Haenszel test for general association for categorical variables, performed for each factor in each comparison. *p<0.05; **Baseline LSM (kPa) thresholds evaluated for statistical significance in the univariate analysis included: <10.7, ≥16.9, and an among-groups comparison of <10.7 vs. ≥10.7 to <16.9 vs. ≥16.9; none reached significance at a level of p<0.05; †Assessed in N=76 patients. ALB, albumin; ALP, alkaline phosphatase; ALT, alanine aminotransferase; AST, aspartate aminotransferase; BMI, body mass index; CBR, composite biochemical response; ELF, Enhanced Liver Fibrosis; GGT, gamma-glutamyl transferase; LSM, liver stiffness measurement; PBC, primary biliary cholangitis; ULN, upper limit of normal.

Table S6. Odds of LSM stability or improvement versus ≥20% worsening at Month 36 by biochemical response at Month 12.

	N	<20% worsening or any improvement (N=78)	≥20% Worsening (N=35)
**Biochemical response at Month 12, n (%)**			
**ALP normalization**	50	39 (78.0)	11 (22.0)
**CBR with ALP >ULN**	43	32 (74.4)	11 (25.6)
**CBR non-response (reference)***	20	7 (35.0)	13 (65.0)
**Odds ratio for LSM stability/improvement** at Month 36 (95% CI)**			
**ALP normalization†**	—	6.58 (2.11, 20.52)	
**CBR with ALP >ULN†**	—	5.40 (1.72, 17.00)	

A logistic regression model with achievement of biochemical response at Month 12 as the fixed effect was used for comparison. *This group included patients who did not achieve CBR or ALP normalization; **Stability/improvement defined as <20% worsening or any improvement in LSM; †Compared with CBR non-response reference group; **This group included patients who did not achieve CBR and did not achieve ALP normalization. ALP, alkaline phosphatase; CBR, composite biochemical response; LSM, liver stiffness measurement; ULN, upper limit of normal.

Table S7. Univariate analysis for factors associated with no LSM increase versus any increase from baseline at Month 36.

	No increase (≤0%) (N=59)	Any increase (>0%) (N=54)	p value
**Baseline characteristics**			
**Age (years), mean (SD)**	59.0 (9.6)	59.4 (9.1)	0.9381
**Age at PBC diagnosis (years), mean (SD)**	48 (8.4)	50 (7.7)	0.2171
**Female, n (%)**	55 (93.2)	51 (94.4)	0.7884
**BMI ≥30 (kg/m^2), n (%)**	24 (40.7)	14 (25.9)	0.0988
**LSM (kPa), mean (SD)****	9.8 (5.5)	8.2 (4.5)	0.0771
**≥10 kPa, n (%)**	19 (32.2)	12 (22.2)	0.2370
**ELF, mean (SD)**	9.8 (0.9)	9.5 (0.8)	0.1002
**AST (U/L), mean (SD)**	33.8 (11.6)	36.3 (14.1)	0.3838
**ALT (U/L), mean (SD)**	39.8 (18.7)	43.2 (22.4)	0.5215
**ALP (U/L), mean (SD)**	244.8 (83.8)	291.8 (104.3)	0.0161*
**<1.5× ULN, n (%)**	13 (22.0)	4 (7.4)	0.0306*
**Total bilirubin (mg/dL), mean (SD)†**	0.6 (0.2)	0.8 (0.3)	0.0477*
**GGT (U/L), mean (SD)**	193.4 (195.4)	234.5 (200.9)	0.0556
**ALB (g/dL), mean (SD)**	4.2 (0.3)	4.2 (0.2)	0.1170
**Platelet (10^3/uL), mean (SD)**	237.6 (82.1)‡	245.8 (62.4)^	0.5931
**Cirrhosis, n (%)**	9 (15.3)	7 (13.0)	0.7283
**On-treatment biochemical response at Month 12**			
**CBR, n (%)**	55 (93.2)	36 (66.7)	0.0004*
**ALP <1.67× ULN, n (%)**	55 (93.2)	41 (75.9)	0.0105*
**ALP ≥15% reduction, n (%)**	58 (98.3)	50 (92.6)	0.1420
**Total bilirubin ≤ULN), n (%)**	59 (100)	49 (90.7)	0.0173*
**ALP <1.5× ULN, n (%)**	51 (86.4)	39 (72.2)	0.0619
**ALP ≤ULN, n (%)**	30 (50.8)	20 (37.0)	0.1416

Statistical differences were assessed using a two-sided Wilcoxon rank sum test for continuous variables and a Cochran-Mantel-Haenszel test for general association for categorical variables, performed for each factor in each comparison. *p<0.05; **Baseline LSM (kPa) thresholds evaluated for statistical significance in the univariate analysis included: <10.7, ≥16.9, and an among-groups comparison of <10.7 vs. ≥10.7 to <16.9 vs. ≥16.9; none reached significance at a level of p<0.05; †Total bilirubin ≤ULN and <0.6× ULN were additionally analyzed and did not reach significance; ‡Assessed in N=58 patients; ^Assessed in N=53 patients. ALB, albumin; ALP, alkaline phosphatase; ALT, alanine aminotransferase; AST, aspartate aminotransferase; BMI, body mass index; CBR, composite biochemical response; ELF, Enhanced Liver Fibrosis; GGT, gamma-glutamyl transferase; LSM, liver stiffness measurement; PBC, primary biliary cholangitis; ULN, upper limit of normal.

Table S8. Odds of no LSM increase versus any increase from baseline at Month 36.

	N	No increase (≤0%) (N=59)	Any increase (>0%) (N=54)
**Biochemical response at Month 12, n (%)**			
**ALP normalization**	50	30 (60.0)	20 (40.0)
**CBR with ALP >ULN**	43	25 (58.1)	18 (41.9)
**CBR non-response (reference)***	20	4 (20.0)	16 (80.0)
**Odds ratio for LSM stability/improvement** at Month 36 (95% CI)**			
**ALP normalization†**	—	6.00 (1.75, 20.59)	
**CBR with ALP >ULN†**	—	5.56 (1.59, 19.43)	

A logistic regression model with achievement of biochemical response at Month 12 as the fixed effect was used for comparison. *This group included patients who did not achieve CBR or ALP normalization; **Stability/improvement defined as no increase in LSM; †Compared with CBR non-response reference group. ALP, alkaline phosphatase; CBR, composite biochemical response; LSM, liver stiffness measurement; ULN, upper limit of normal.

SUPPLEMENTARY REFERENCES

1. Bowlus CL, Galambos MR, Aspinall RJ, et al. A phase II, randomized, open-label, 52-week study of seladelpar in patients with primary biliary cholangitis. J Hepatol 2022;77(2):353–364.
2. Hirschfield GM, Shiffman ML, Gulamhusein A, et al. Seladelpar efficacy and safety at 3 months in patients with primary biliary cholangitis: ENHANCE, a phase 3, randomized, placebo-controlled study. Hepatology 2023;78(2):397–415.
3. Levy C, Trivedi PJ, Kowdley KV, et al. Long-Term Efficacy and Safety of Selective PPARδ Agonist Seladelpar in Primary Biliary Cholangitis: ASSURE Interim Study Results. Am J Gastroenterol 2025;121(5):1140–1153:Suppl. Epub ahead of print.
